# Achieving High Damping Capacity in Oxygen‐Enhanced BCC Zr‐Hf‐Ti‐Nb Multi‐Principal‐Element Alloys with Low Young's Modulus

**DOI:** 10.1002/advs.202501068

**Published:** 2025-04-29

**Authors:** Qing Wang, Zhenhua Wang, Qixiang Zhang, Rui Wang, Tongmin Wang, Chaoli Ma, Ang Li, Xiaodong Han, Junhua Luan, Zengbao Jiao, Peter K. Liaw

**Affiliations:** ^1^ School of Materials Science and Engineering Key Laboratory of Materials Modification by Laser Ion and Electron Beams (Ministry of Education) Dalian University of Technology Dalian 116024 China; ^2^ Tianmushan Laboratory Yuhang District Hangzhou 310023 China; ^3^ Beijing Key Laboratory of Microstructure and Property of Advanced Materials Faculty of Materials and Manufacturing Beijing University of Technology Beijing 100124 China; ^4^ Inter‐University 3D Atom Probe Tomography Unit Center for Advanced Nuclear Safety and Sustainable Development City University of Hong Kong Hong Kong 999077 China; ^5^ Department of Mechanical Engineering Research Institute for Advanced Manufacturing The Hong Kong Polytechnic University Hong Kong 999077 China; ^6^ Department of Materials Science and Engineering University of Tennessee Knoxville TN 37996 USA

**Keywords:** multi‐principal‐element alloys, BCC structural stability, mechanical property, damping capacity, Snoek‐type relaxation

## Abstract

Multi‐principal‐element alloys (MPEAs) have gained widespread popularity due to the efficient synergetic regulation of mechanical and functional properties in a huge compositional space. Here, novel O‐enhanced BCC Zr‐Hf‐Ti‐Nb MPEAs with prominent mechanical and damping properties are developed by the composition formula of (Zr,Hf,Ti)_15_Nb_3_. The Zr_14_TiNb_3_ and Zr_8_Hf_6_TiNb_3_ alloys possess low BCC‐β structural stability. While the Zr_8_Hf_4_Ti_3_Nb_3_ alloy has a much higher BCC‐β stability, as evidenced by the fact that only few α'' and ω precipitates appear in 1.8 at% oxygen‐added alloy. This alloy exhibits an optimal mechanical property with a higher yield strength (*σ*_YS_ = 1000 MPa) and larger ductility (*ε* = 15.1%), which is ascribed to the formation of O‐rich clusters in BCC matrix. Moreover, these oxygen‐free and ‐added alloys exhibit an excellent damping capacity due to their low Young's modulus (*E* < 70 GPa), as exemplified with a peak value of (tan*δ*)_max_ = 0.02 for 1.8 at% oxygen‐added alloy. Notably, the damping characteristics are prominent over a wide temperature range (550–800 K), which derives from the occurrence of multiple separated oxygen‐rich clusters. The present findings provide an avenue to enhance mechanical and functional performances of high‐temperature damping alloys.

## Graphical abstract

A series of oxygen‐enhanced Zr‐Hf‐Ti‐Nb MPEAs with high strength and outstanding damping capacity are developed through tailoring the BCC structural stability by an elaborate composition regulation. Strengthening mechanism for high strength and damping mechanism related to various Snoek‐type relaxations reveals that the existence of multiple separate O‐rich clusters is crucial for the improvement of properties.

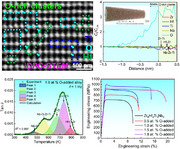

## 1 Introduction

Multi‐principal‐element alloys (MPEAs), also termed as high‐entropy and medium‐entropy alloys, have been enjoying great popularity because their broad composition space can provide great opportunities to realize the synergetic control of multiple properties, including mechanical and functional (corrosion/oxidation‐resistant, irradiation‐resistant, magnetic, thermoelectric, damping, etc.) properties.^[^ ^1^, ^2^, ^3^, ^4^, ^5^, ^6^, ^7^, ^8^, ^9^, ^10^, ^11^, ^12^, ^13^ ^]^ This entropy‐regulated alloying strategy makes MPEAs fill the gap of unexplored space with traditional alloys. Moreover, they still exhibit simple crystalline structures, similar to those of traditional alloys.^[^ ^1^, ^2^, ^3^, ^4^ ^]^ Latest research reported that the fascinating mechanical and soft‐magnetic properties of Al/Ta‐containing Co‐Fe‐Ni‐based MPEAs are ascribed to the coherent microstructure of paramagnetic L1_2_ nanoparticles in the ferromagnetic face‐centered‐cubic (FCC) matrix or ferromagnetic body‐centered‐cubic (BCC) nanoprecipitates in the B2 matrix.^[^ ^8^, ^9^, ^10^, ^11^ ^]^ Also, these soft‐magnetic MPEAs possess outstanding electromagnetic wave absorption properties due to their great superiority in balancing the key characteristic parameters of permittivity and permeability.^[^ ^12^, ^13^ ^]^ Compared with the FCC‐based MPEAs primarily consisting of late transition metals (LTMs), the equimolar or near‐equimolar mixing of early transition metals (ETMs = Ti/Zr/Hf, V/Nb/Ta, and Cr/Mo/W) renders the MPEAs with the BCC solid solution structure.^[^ ^14^, ^15^ ^]^ They are often named as refractory high‐entropy alloys since they generally show high mechanical strength at much higher temperatures.^[^ ^16^, ^17^, ^18^ ^]^ The second‐phase precipitation by the addition of a small amount of Al or Si into the BCC matrix could further enhance the strength of these alloys.^[^ ^14^, ^19^, ^20^, ^21^ ^]^ In addition, these BCC‐based MPEAs are good candidates as energetic structural materials due to their high combustion calorific capacity and superior penetration performance triggered by self‐sharpening behavior, such as HfZrTiTa_0.53_, Al_2_Ti_6_Zr_2_Nb_3_Ta_3_, and WMoNiFe.^[^ ^22^, ^23^, ^24^ ^]^ The interstitial oxygen or nitrogen doping in Ti‐Zr‐Hf‐Nb‐Ta‐based MPEAs not only enhances their mechanical properties with higher strength and larger ductility, but also exhibits an excellent damping capacity induced by point defects, without changing the BCC solid solution structure.^[^ ^25^, ^26^ ^]^

It is emphasized that in these BCC‐based MPEAs, the equimolar mixing of each element can induce a BCC phase separation into two BCC phases, i.e., the Ti/Zr‐rich BCC1 and Mo/Ta/Nb‐rich BCC2, resulting in obvious embrittlement of alloys.^[^ ^27^ ^]^ It is mainly ascribed to the excessive addition of BCC‐stabilizers of Mo, Nb, Ta, etc. into the Ti/Zr/Hf matrix. Otherwise, the BCC phase would be destabilized and some other metastable phases (such as α‘, α'’, and ω) could precipitate from the BCC matrix if the amount of Mo/Nb/Ta is insufficient, leading to a change in mechanical properties.^[^ ^27^, ^28^, ^29^ ^]^ Also, the interstitial oxygen atoms can destabilize the BCC structure to a certain extent and promote the precipitation of brittle ω phase, which is detrimental to the ductility.^[^ ^30^, ^31^, ^32^ ^]^ Noticeably, both the BCC phase separation and the precipitation of metastable phases (especially the ω) can affect the Young's modulus (*E*) of BCC β‐Ti and β‐Zr alloys in the form of an increasing tendency.^[^ ^29^, ^32^, ^33^, ^34^ ^]^ However, a lower *E* is necessary for the high‐damping alloys to eliminate noise and mechanical vibrations, since the ratio of loss modulus to storage modulus, i.e., *tanδ*, represents the damping capacity under the condition that the internal friction is anelastic.^[^ ^35^, ^36^ ^]^ A typical BCC‐β Ti‐25Nb‐1.5O (atomic percent, at%) alloy with a low *E* of 65 GPa has a much higher damping capacity with a peak value of *tanδ* = 0.033 at 489 K at the frequency of *f* = 1 Hz, which is attributed mainly to the Snoek‐type strain relaxation caused by the reorientation of interstitial oxygen atoms in BCC solid solution.^[^ ^37^ ^]^ Actually, the damping capability is related to the mobile defects, while the high strength originates from the immobile defects.^[^ ^35^, ^36^, ^37^, ^38^ ^]^ This trade‐off could be well optimized in multi‐component systems.^[^ ^3^, ^26^ ^]^ For example, a high damping capacity with a peak value of *tanδ* = 0.02 at 747 K (*f* = 1 Hz) and a high yield strength of 1138 MPa are achieved simultaneously in the single‐phase BCC‐TiZrHfNb_0.5_Ta_0.5_ high‐entropy alloy doped by a minor amount of 2.0 at% oxygen addition, in which the damping capability is dependent on the mobile oxygen atoms and the strength is enhanced by the oxygen ‐ordered interstitial complexes.^[^ ^26^ ^]^ Besides, the Zener‐type relaxation induced by substitutional atoms also makes an important contribution to the damping behavior, as evidenced by the fact that the damping capacity of BCC TiZrHfNb_0.5_Ta_0.5_ alloy is significantly higher than that of Ti‐25 at%Nb alloy.^[^ ^26^, ^37^ ^]^

It is known that the Snoek‐type relaxation process is closely related to the migration of interstitial oxygen atoms in BCC host lattice under an external cycle stress, during which a large amount of oxygen can produce a higher damping capacity.^[^ ^35^, ^36^, ^37^, ^39^ ^]^ However, it is difficult to occur for the Snoek effect in α'' and ω phases since they have orthorhombic or/and close‐packed hexagonal structures that reduces drastically the oxygen content for relaxation.^[^ ^35^, ^40^ ^]^ Moreover, the segregation of interstitial oxygen atoms at the interface between the α''/ω and BCC‐β phases weakens the relaxation‐induced damping capability.^[^ ^41^ ^]^ Thus, the BCC‐β structural stability is primarily required for this kind of high‐damping alloys, which is contradictory with the fact that the oxygen addition destabilizes the BCC‐β structure to a certain extent and promotes the precipitation of brittle ω phases. So, it is of great importance to optimize alloying additions (substitutional and interstitial elements) rationally for obtaining a stable BCC‐β structure. More expectantly, the BCC‐β phase should be stabilized at the lower limit to avoid both the second‐phase precipitation and phase separation of the BCC matrix, at which a much lower *E* would be achieved resultantly.

In our previous works, we designed a series of Zr‐base medium‐entropy alloys with the composition of [Ti‐(Zr,Hf,Ti)_14_]Nb_3_ (= (Zr,Hf,Ti)_15_Nb_3_) via the cluster formula approach for tailoring the BCC‐β structural stability (see details in “Composition design via the cluster formula approach” section of Supporting Information).^[^ ^29^ ^]^ It was found that the sufficient amount of Nb addition enables these Zr‐base alloys dominated by the BCC structure, which can avoid the formation of α phase, as evidenced by the fact that the Zr_14_Nb_3_ alloy possesses a primary BCC‐β structure with a minor amount of ω precipitation. And an appropriate amount of Ti and Hf substitution for Zr can further enhance the BCC‐β structural stability. Notably, the single BCC‐β structure appears in the [Ti‐Zr_14_]Nb_3_ (= Zr_14_TiNb_3_) alloy, exhibiting the lowest Young's modulus of *E* = 57 GPa in the as‐cast state. However, the further substitution of Ti for Nb will deteriorate the BCC‐β stability of Zr_14_Ti_2_Nb_2_ and Zr_14_Ti_3_Nb_1_ alloys due to the precipitation of a large amount of ω nanoparticles. And the complete substitution of Hf for Zr also weakens the BCC stability of Hf_14_Ti_1_Nb_3_ alloy, as evidenced by a large amount of ω nanoprecipitates in the BCC matrix. By contrast, the partial substitution of Hf for Zr slightly reduces the BCC‐β stability of [Ti‐Zr_8_Hf_6_]Nb_3_ (= Zr_8_Hf_6_TiNb_3_) alloy due to a minor amount of ω precipitation. Based on it, the further replacement of Ti for Hf can render the [Ti‐Zr_8_Hf_4_Ti_2_]Nb_3_ (= Zr_8_Hf_4_Ti_3_Nb_3_) alloy with a single BCC‐β structure. Furthermore, the Young's modulus of these two alloys are *E* ∼ 70 GPa, which indicates that both the precipitation of ω and a much higher β stability can increase the *E*.^[^ ^29^ ^]^ Since these three designed BCC MPEAs (Zr_14_TiNb_3_, Zr_8_Hf_6_TiNb_3_, and Zr_8_Hf_4_Ti_3_Nb_3_) have relatively lower *E*, which is necessary for a high damping capacity, the present work will investigate the influence of the addition of interstitial oxygen on the BCC‐β structural stability, mechanical property, and damping capacity of these MPEAs. Here, all the designed alloys with and without the oxygen additions will be homogenized first. And the microstructural characterization, tensile property, and damping property measurements will be then conducted. Finally, the related mechanisms for high strength and high damping capacity of these alloys will be discussed, with an emphasis on the relationship between the microstructure and macroscopic properties.

## 2 Results

### 2.1 Microstructural Characterization of MPEAs with and without O Additions

**Figure 1:**
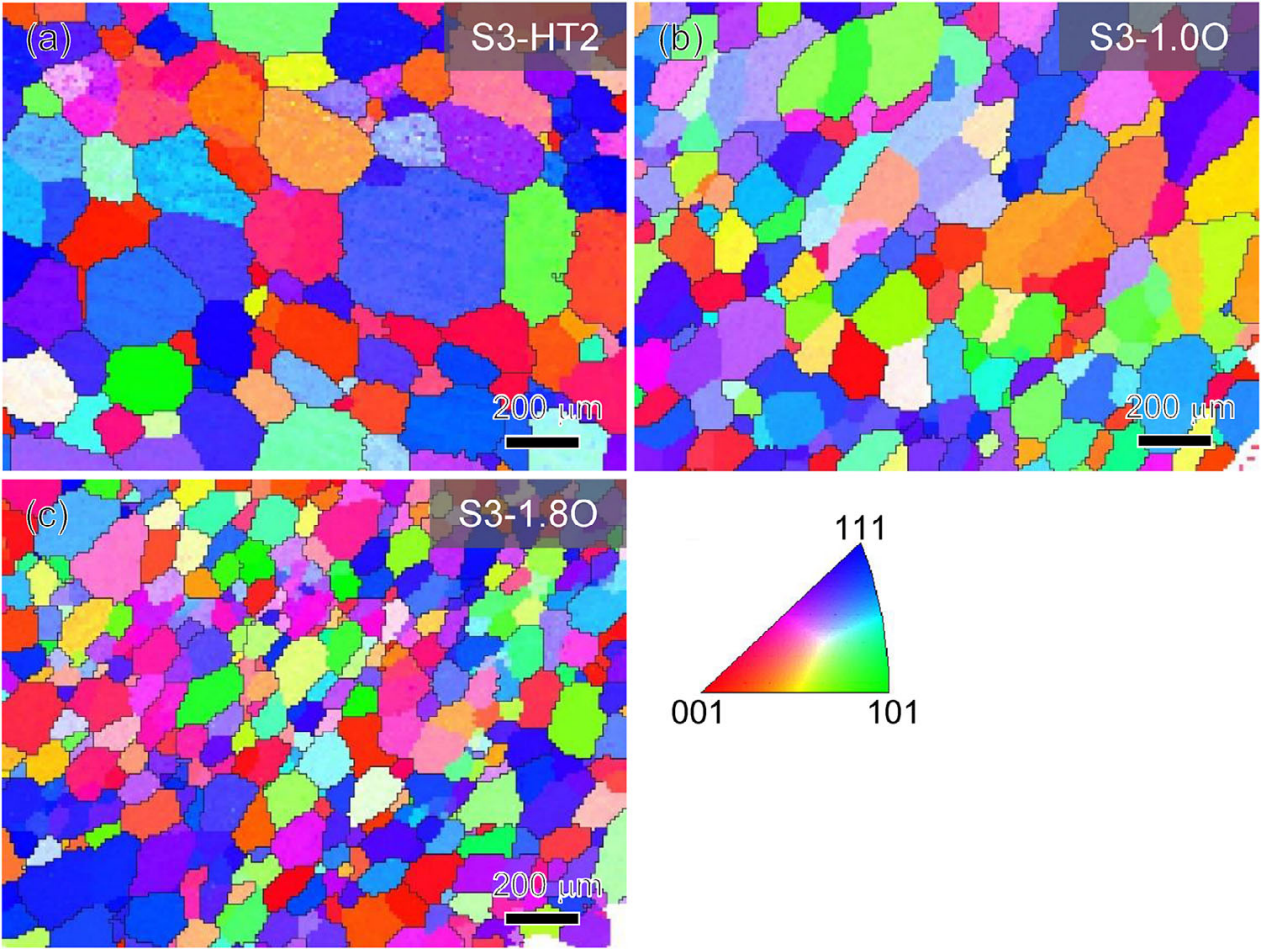
EBSD‐IPF maps of the homogenized S3‐HT2 alloys containing different O contents. a) S3‐HT2, b) S3‐1.0O, and c) S3‐1.8O.

The X‐ray diffractometer (XRD) patterns of Zr_14_TiNb_3_ (labeled as S1‐T), Zr_8_Hf_6_TiNb_3_ (S2‐HT1), Zr_8_Hf_4_Ti_3_Nb_3_ (S3‐HT2), and their O‐added alloys with different O contents (0.2–1.8 at%) after the homogenized treatment in Figure S1 (Supporting Information), reveal that all these MPEAs possess a single‐phase BCC solid solution structure, without the detection of other diffraction peaks. The lattice constant (*a*) values of the BCC phase in these alloys are listed in Table S1 (Supporting Information), where the chemical compositions in atomic percent (at%) are also included. Microstructures of all these homogenized alloys with and without O additions were characterized by optical microscopy (OM) and electron backscatter diffraction (EBSD). The OM observations indicate that the O‐free alloys have a fully‐recrystallized equiaxial microstructure with an average grain size of 100–130 µm, as shown in Figure S2 (Supporting Information). **Figure** **1** shows the EBSD inverse pole figure (IPF) maps of the homogenized S3‐HT2 alloys containing different O contents. It can be seen that these alloys also exhibit equiaxed grains, but the grain size is reduced by the O, as exampled by the grain size of S3 and S3‐1.8O alloys being ≈120 and ≈70 µm, respectively. It implies that the addition of O can refine the grains.

**Figure 2:**
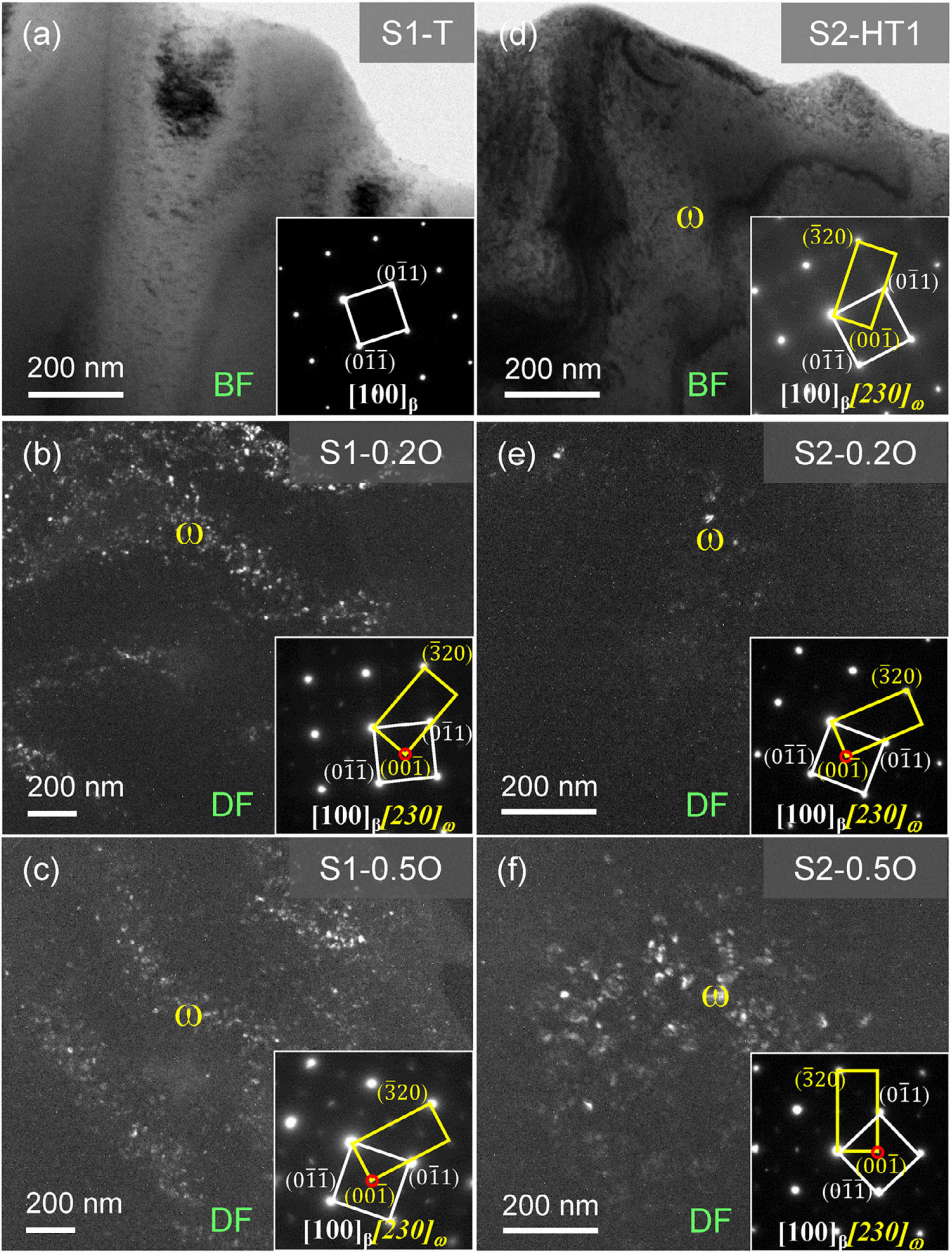
TEM characterization of S1‐T and S2‐HT1 alloys containing different O content. a) BF‐TEM image and corresponding SAED pattern along the [100] direction of S1‐T alloy, exhibiting a single BCC solid solution phase; b,c) DF‐TEM images and corresponding SAED patterns of the b) S1‐0.2O and c) S1‐0.5O alloys; d–f) BF‐ and DF‐TEM images and corresponding SAED patterns of the d) S2‐HT1, e) S2‐0.2O, and f) S2‐0.5O alloys. These results show that the O addition accelerates the precipitation of ω nanoparticles from the BCC‐β matrix.

After homogenization, the S1‐T alloy exhibits a single BCC‐β solid solution structure, without any other metastable phases, as shown in bright‐field (BF)‐transmission electron microscope (TEM) image (**Figure** **2**a), indicating that this alloy has a high enough BCC‐β stability. However, when a minor amount (0.2–0.5 at%) of O was added, ω nanoparticles precipitate from the BCC‐β matrix, as identified by the dark‐field (DF)‐TEM images and corresponding selected‐area electron diffraction (SAED) patterns in Figure 2b,c. It is easy to realize the phase transformation from the BCC‐β to ω by the collapse of {222}_β_ planes to intermediate positions within β lattice structure, resulting in the crystallographic relationship characterized by {111}<110>_β_ // (001)<110>_ω_.^[^ ^29^, ^42^, ^43^ ^]^ It demonstrates that the O element destabilizes the BCC‐β structure and results in the second‐phase precipitation.^[^ ^30^, ^31^, ^32^ ^]^ The substitution of Hf for Zr slightly weakens the BCC‐β structural stability due to the formation of a minor amount of ω nanoparticles in the BCC matrix of the S2‐HT1 alloy (Figure 2d). The doping of O induces the precipitation of ω phase, as seen in Figure 2e,f, following a similar tendency to that in S1‐T.

**Figure 3:**
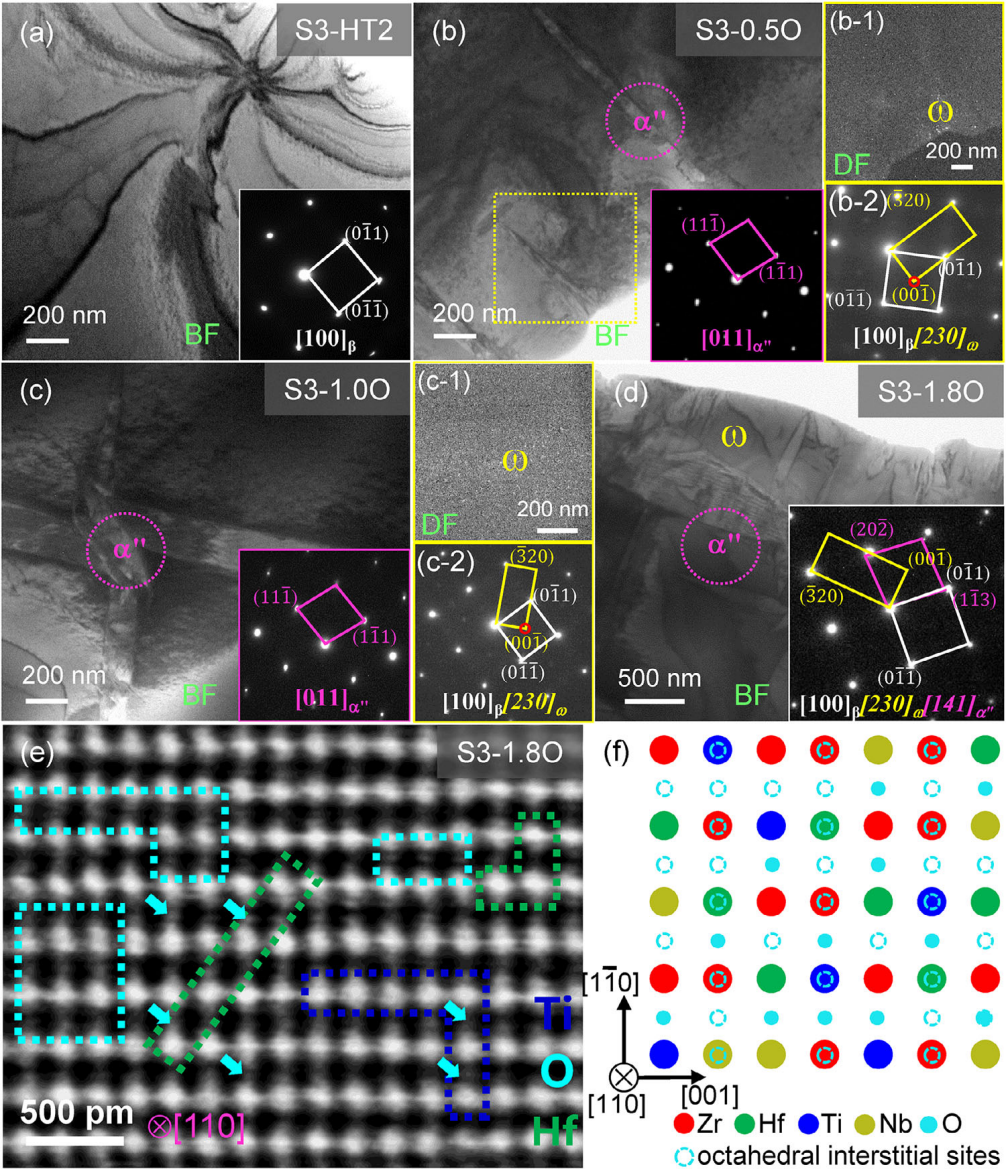
TEM characterization of the O‐free and O‐added S3‐HT2 alloys. a) BF‐TEM image and corresponding SAED pattern along the [100] direction of O‐free S3‐HT2 alloy, showing a single BCC phase structure; b–d) BF‐ and DF‐TEM images, as well as the corresponding SAED patterns of S3‐0.5O b), S3‐1.0O c), and S3‐1.8O d) alloys, exhibiting the precipitation of α“” laths and few ω nanoparticles in BCC matrix. e) The iDPC‐STEM image along the [110] direction in S3‐1.8O alloy, where the dark regions enriched in light Ti atoms and the bright regions enriched in heavy Hf atoms in the BCC lattice are marked with the blue and green rectangles, respectively. O atoms occupy the octahedral interstitial sites in the BCC lattice, as labeled by the cyan arrows and rectangles, indicating the formation of O‐rich clusters. Moreover, the schematic diagram of the BCC lattice structure containing Zr, Hf, Ti, Nb, and O atoms is displayed in f), in which the octahedral interstice sites are projected along [110] direction and O‐rich clusters are well presented.

By contrast, the S3‐HT2 (Zr_8_Hf_4_Ti_3_Nb_3_) alloy contains very small amounts of ω nanoparticles, together with some α'' laths (**Figure** **3**a,b) after the addition of 0.5 at% O, demonstrating a much higher BCC‐β structural stability. It is fascinating that the O content in this alloy can reach up to 1.8 at%, and it is more likely to form α'' laths than ω nanoparticles, as shown in the samples with 1.0–1.8 at% O (Figure 3c,d). Since the precipitation of α'' from the BCC‐β matrix is competitive with that of ω during quenching,^[^ ^42^, ^43^ ^]^ the formation of α'' laths inevitably results in a reduction of ω particles, which is commonly observed in metastable Ti‐ and Zr‐base alloys.^[^ ^44^, ^45^, ^46^, ^47^ ^]^ Moreover, the phase compositions of α'' and ω are very similar to that of the BCC‐β, as evidenced by the elemental distributions (Figure S3, Supporting Information) in S3‐1.8O alloy mapped with electronic probe micro‐analyzer (EPMA), showing that the constituent elements are uniformly distributed within the matrix grains. Besides, the α'' laths do not induce embrittlement but increases the Young's modulus slightly in both multi‐component β‐Ti and β‐Zr alloys.^[^ ^32^ ^]^ Here, it needs to be emphasized that when the O content was further added into these alloys, the alloys become very brittle due to the precipitation of a large amount of brittle ω particles. In addition, Table S2 (Supporting Information) gives the measured compositions of these designed alloys, which are consistent with their nominal ones. And even in the O‐free S3‐HT2 alloy, the oxygen content is ≈0.09 at%, which results from the fabrication process.

To characterize the atomic‐scale structure of O‐added S3‐HT2 alloys, we employed the state‐of‐the‐art integrated Differential Phase Contrast scanning TEM (iDPC‐STEM) technique, which possesses the advantages of high‐angle annular dark field (HAADF) and annular bright field (ABF) techniques and can image light and heavy elemental atoms together.^[^ ^48^ ^]^ Figure 3e shows the iDPC‐STEM image along the [110] direction of S3‐1.8O alloy, in which there exist dark regions enriched in light Ti atoms (or Ti‐rich clusters, marked with blue rectangles) and bright regions segregated by heavy Hf atoms (Hf‐rich clusters, marked with green rectangles) in the BCC lattice sites, since the Z‐contrast is greatly sensitive to the local compositional variations in the atomic number of constituent elements in a specific atomic column.^[^ ^49^ ^]^ This observation demonstrates the formation of chemical short‐range orders (CSROs) in this alloy due to the locally‐heterogeneous distribution of elemental atoms, which is the intrinsic feature of BCC‐based MPEAs.^[^ ^25^, ^26^, ^50^ ^]^ More importantly, the O atoms occupy the octahedral interstices in the BCC lattice of S3‐1.8O alloy, as labeled by the cyan arrows and rectangles in Figure 3e. To clarify more clearly, the schematic diagram of the BCC lattice structure containing Zr, Hf, Ti, Nb, and O atoms is shown in Figure 3f, where the octahedral interstice sites are projected along the [110] direction. It is found that O atoms prefer to occupy in the octahedral interstices adjacent to Hf‐ and Ti‐rich regions, forming Hf‐O‐ and Ti‐O‐rich clusters, respectively. Besides, other O atoms are inevitably located in the interstitial sites near the substitutional Zr and Nb atoms to form Zr‐O and Nb‐O segregations. Experimentally, similar phenomenon of O‐rich clusters also occurs in the S3‐1.0O alloy in spite of its lower O content (1.0 at%), as identified by iDPC‐STEM image in Figure S4 (Supporting Information).

**Figure 4:**
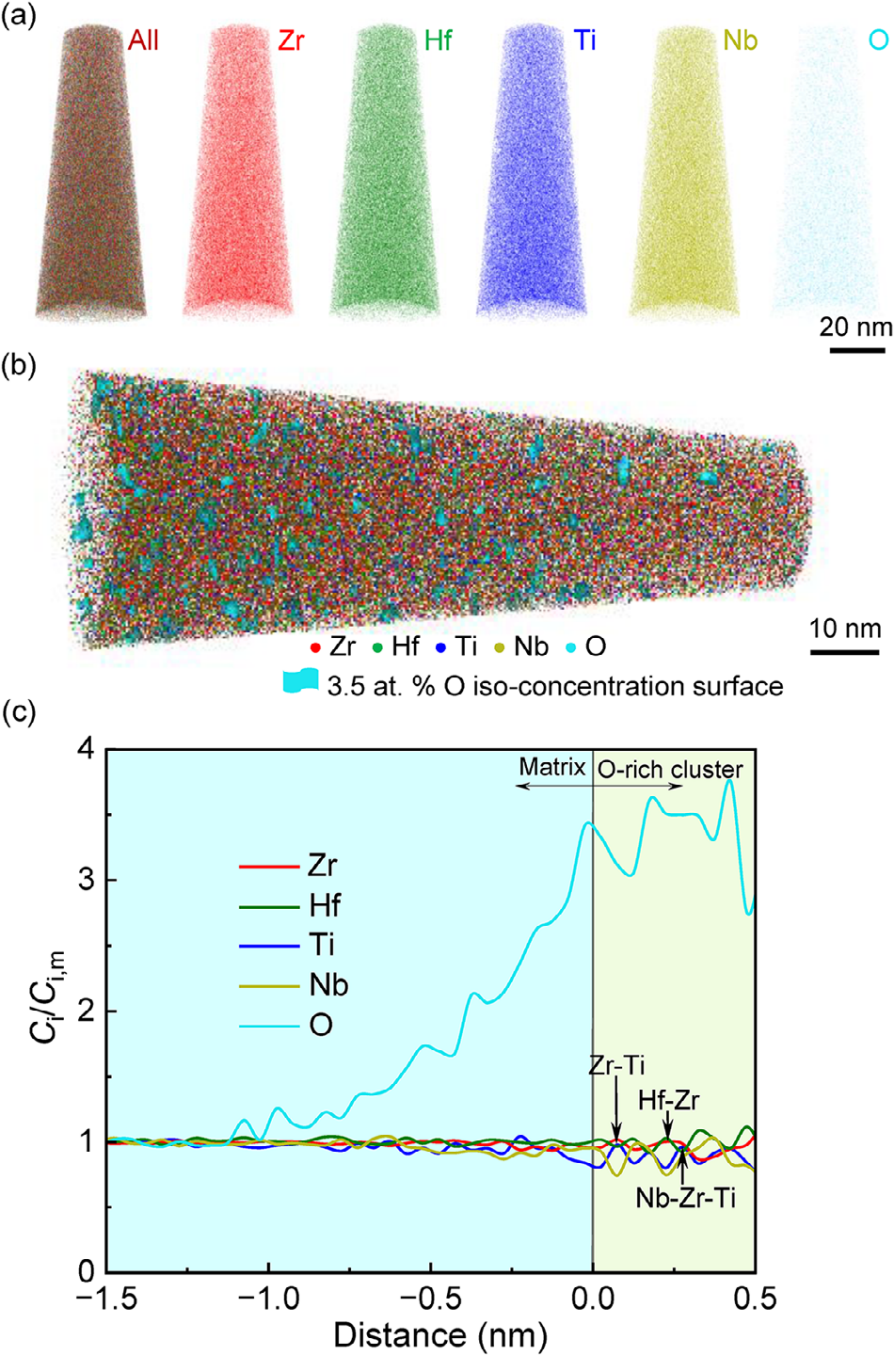
APT characterization of the S3‐1.8O alloy. a) Atom maps of Zr, Hf, Ti, Nb, and O; b) The concentration isosurface of 3.5 at% O, showing the existence of O‐rich clusters; and c) The corresponding profile of the ratio of the elemental composition to its respective matrix composition as a function of the distance to the concentration isosurface of 3.5 at% O, demonstrating that there exist diverse concentration fluctuations in Zr, Ti, Hf, and Nb. Complex O‐rich clusters are also marked by arrows.

The atom probe tomography (APT) characterization was further performed to study the O‐rich clusters in S3‐1.8O alloy. **Figure** **4**a presents the elemental mapping of Zr, Hf, Ti, Nb, and O, in which the positions and extents of Zr (red), Hf (green), Ti (blue), Nb (yellow), and O (cyan) are indicated. For statistical analysis, the cyan concentration isosurface of 3.5 at% O is shown in Figure 4b to visualize the O‐rich clusters. The proximity histogram concentration profiles are shown in Figure S5 (Supporting Information) as a function of distance to the isosurfaces, which exhibits the elemental partitioning behavior across the interface between the matrix and O‐rich clusters. It is found that there exists a significant fluctuation in the elemental concentration of Zr, Ti, Hf, Nb, and O, especially within the O‐rich cluster. To demonstrate the solute partitioning more clearly, Figure 4c gives the variation of the ratio of the composition of each element to their respective matrix composition with the distance to the isosurface. It can be seen that near the interface between the matrix and O‐rich clusters, the concentration of Ti decreases drastically, while that of Hf rises slowly along with the significant increase in the O concentration, besides a slight fluctuation in the Zr and Nb concentration distributions. It implies the formation of Hf‐O‐rich clusters, which can be corresponding to the bright regions segregated by heavy Hf atoms in the iDPC‐STEM observation (Figure 3e). In the O‐rich clusters, there exist different fluctuations in the concentrations of Zr, Ti, Hf, and Nb, which leads to form alternating regions of elemental enrichment and deficiency, representing simple Zr‐O‐, Hf‐O‐, Ti‐O‐, and Nb‐O‐rich clusters, respectively. These observations are consistent with the iDPC‐STEM results. Moreover, two or three kinds of elements can occur simultaneously at the same position in some regions due to the periodicity of concentration fluctuations, as marked by the arrows in Figure 4c, indicating the formation of multiple complex clusters, such as Zr‐Ti‐O‐, Hf‐Zr‐O‐, and Nb‐Zr‐Ti‐O‐rich clusters.

### 2.2 Mechanical Properties

**Figure 5:**
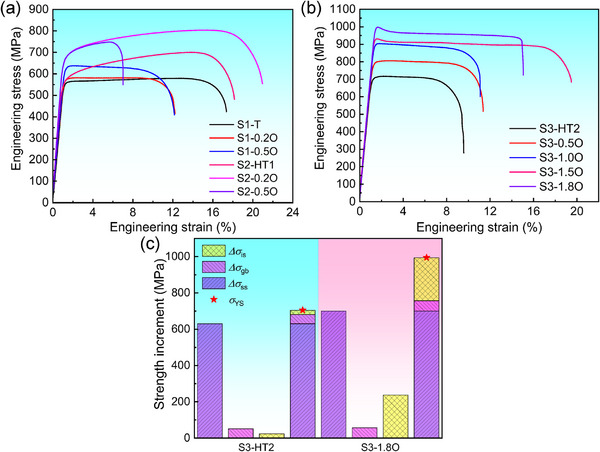
Mechanical properties of the designed alloys. Tensile engineering stress‐strain curves of the a) S1‐T and S2‐HT1 alloys containing 0–0.5 at% O; and b) S3‐HT2 alloys containing 0–1.8 at% O. c) Calculated strength increments from different strengthening mechanisms in O‐free and 1.8 at% O‐containing S3‐HT2 alloys, in which the measured yield strength values are also presented with red stars for comparison.

The tensile engineering stress‐strain curves of the Zr_14_TiNb_3_ (S1‐T), Zr_8_Hf_6_TiNb_3_ (S2‐HT1), and Zr_8_Hf_4_Ti_3_Nb_3_ (S3‐HT2) containing different O contents (0–1.8 at%) are shown in **Figure** **5**, from which the mechanical properties, including yield strength (*σ*_YS_), ultimate tensile strength (*σ*_UTS_), elongation to fracture (*ε*), and Young's modulus (*E*), are listed in Table S1 (Supporting Information). By comparing these three O‐free alloys, it can be found that the co‐alloying of multi‐principal elements can improve the mechanical properties of the alloys, as evidenced by the enhanced yield strength from *σ*_YS_ = 554 MPa of S1‐T to *σ*_YS_ = 704 MPa of S3‐HT2 due to the solid‐solution strengthening of substitutional atoms. A trace amount of O addition into the S1‐T and S2‐HT1 enhances the mechanical strength obviously, as exemplified by the fact that the *σ*_YS_ = 563 MPa of S2‐HT1 increases to *σ*_YS_ = 670 MPa after the minor‐alloying by 0.5 at% O (Figure 5a). It can be mainly ascribed to the solid‐solution strengthening of interstitial O atoms and the precipitation of ω nanoparticles. However, the ductility is reduced sharply from *ε* = 17.4% of O‐free alloy to *ε* = 6.2% of S2‐0.5O, which should be ascribed to the precipitation of brittle ω phase.^[^ ^29^, ^32^ ^]^

Fascinatingly, the further addition of O into the S3‐HT2 alloy can improve the strength and ductility simultaneously, as presented in Figure 5b. The yield strength is enhanced continuously from *σ*_YS_ = 704 MPa without O to 796 MPa with 0.5 at% O, 896 MPa with 1.0 at% O, and then to 1000 MPa with 1.8 at% O. Meanwhile, the elongation also increases from original *ε* = 9.2% to 15.1–18.6% (for alloys containing 1.5–1.8 at% O). For the Young's modulus of these MPEAs, the S1‐T alloy has the lowest modulus of *E* = 49 GPa. Both the precipitation of a minor amount of ω and the increase in the BCC‐β stability enhance the Young's moduli of S2‐HT1 and S3‐HT2 alloys slightly, being about *E* ∼ 62 GPa. The O addition does not increase the modulus of alloys sharply, as evidenced by *E* = 68 GPa of S3‐1.8O alloy, which is mainly attributed to that the amount of O‐induced ω precipitates is still at a lower level. Thus, all these alloys exhibit a lower Young's modulus with *E* < 70 GPa. When an excessive amount (2.5 at%) of O was added into S3‐HT2 alloy, a large amount of ω nanoparticles could precipitate from the BCC‐β matrix, as shown in Figure S6a (Supporting Information). Consequently, this alloy possesses a higher modulus of *E* = 78 GPa and a limited ductility of *ε* = 3.2%, despite the higher strength of *σ*_YS_ = 1013 MPa (Figure S6b, Supporting Information). Actually, it has been extensively demonstrated that a moderate O content (1.0–2.0 at%) is optimal for the BCC‐β Zr‐Hf‐Ti‐Nb alloys, as it can maintain both ductility and Young's modulus, which is primarily determined by the BCC structural stability.^[^ ^32^, ^46^, ^51^, ^52^, ^53^, ^54^ ^]^ Thus, the O content of 1.8 at% may be the optimal for the S3‐HT2 alloy, and an excessive O addition (> 2.0 at%) will sacrifice the ductility and modulus due to the formation of a large amount of ω and α phases, despite resulting in higher strength.

### 2.3 Damping Properties of Zr_8_Hf_4_Ti_3_Nb_3_ with and without O Content

**Figure 6:**
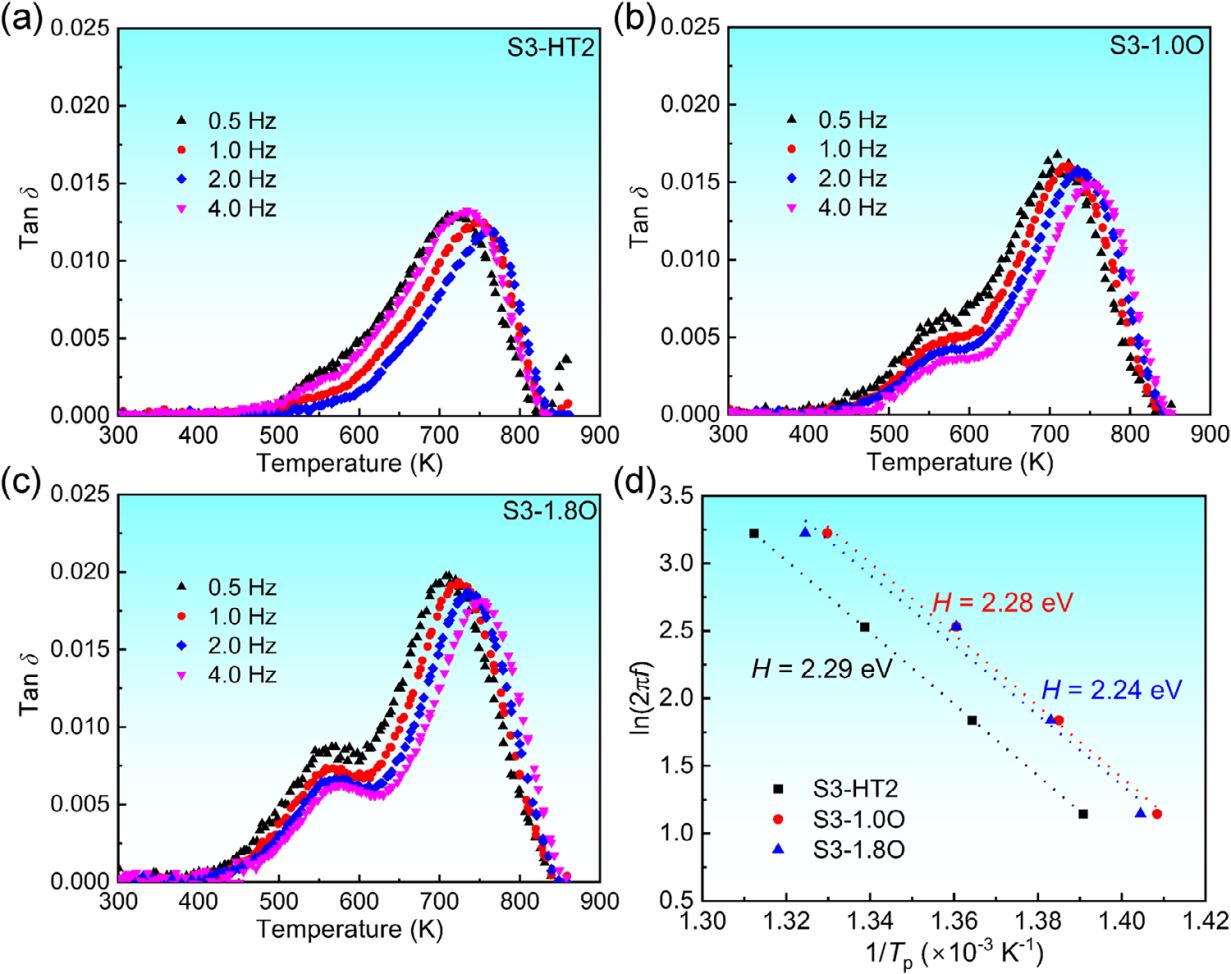
Damping properties of the O‐free and O‐added S3‐HT2 alloys. Temperature‐dependent variation of the damping capacity (tan*δ*) in a) S3‐HT2, b) S3‐1.0O, and c) S3‐1.8O alloys. The variations of ln(2*πf*) with 1/*T_p_* in these alloys were plotted in d), which exhibits a linear relationship, and the slope represents the activation energy *H* for the stress‐induced reorientation process.

Since the S3‐HT2 (Zr_8_Hf_4_Ti_3_Nb_3_) alloys containing different O contents exhibit outstanding mechanical properties with high strength and large ductility, the O‐free and O‐containing alloys with 1.0 at% O and 1.8 at% O were selected to investigate their damping behaviors. Here, the damping capacity was characterized by the parameter of *tanδ*, which is the ratio of the loss modulus to storage modulus for representing the energy dissipation in alloys.^[^ ^35^, ^36^ ^]^ **Figure** **6** shows the temperature‐dependent variations of *tanδ* of these three alloys by the forced vibration at *f* = 0.5, 1.0, 2.0, and 4.0 Hz, respectively. The measured damping peak (*tanδ*)_max_ indicates the maximum energy dissipation during the damping process, where the alloys with (*tanδ*)_max_ ≥ 0.01 are regarded as high‐damping materials.^[^ ^35^ ^]^ It is found that the (*tanδ*)_max_ values of these alloys are all higher than 0.01, showing a better damping ability. Moreover, the damping peak of each alloy could shift to a higher temperature with increasing the applied frequency, which illustrates that the damping is controlled by the strain relaxation induced by the reorientation of point defects (especially the interstitial atoms).^[^ ^35^, ^36^, ^37^ ^]^ In particular, the (*tanδ*)_max_ values at 1.0 Hz in Zr_8_Hf_4_Ti_3_Nb_3_, 1.0 at% O and 1.8 at% O‐added alloys are 0.014, 0.016, and 0.02, respectively (Figure 6a–c), and the corresponding peak temperatures in damping curves are *T_p_* = 723–733 K.

Notably, the strain relaxation in these alloys is more closely related to the reorientation process of interstitial atoms, i.e., the Snoek‐type damping.^[^ ^35^ ^]^ According to this kind of damping mechanism, the energy *H* to activate interstitial atoms for switching their positions is proportional to the peak temperature *T_p_*,^[^ ^37^ ^]^ following the Arrhenius Equation (1):^[^ ^55^ ^]^

(1) $$\ln2\pi f+\ln\tau_{0}+\left(\frac{H}{k_{B}}\right)\;T_{p}^{-1}=0$$

where *f* is the vibration frequency, *τ_0_* is the relaxation time at infinite temperature for the strain relaxation process, and *k_B_* = 1.380649 × 10^−23^ J K^−1^is the Boltzmann constant. Figure 6d plots the variation of ln(2*πf*) with 1/*T_p_*, manifesting a linear relationship. The slope of the relationship represents the activation energy, which was deduced as *H* = 2.29, 2.28, and 2.24 eV for S3‐HT2, S3‐1.0O, and S3‐1.8O alloys, respectively. Compared with simple low‐*E* and BCC β‐Ti alloys ((*tanδ*)_max_ = 0.006 and *T_p_* = 572 K for Ti‐25Nb, (*tanδ*)_max_ = 0.033 and *T_p_* = 489 K for Ti‐25Nb‐1.5O at%),^[^ ^37^ ^]^ the multi‐component alloying with both substitutional and interstitial elements can obviously improve the damping capacity of alloys. It was also identified by (*tanδ*)_max_ = 0.014–0.02 and *T_p_* = 730–750 K in TiZrHfNb_0.5_Ta_0.5_ alloy with 2 at% O addition.^[^ ^26^ ^]^

Besides the damping peak (*tanδ*)_max_ and the peak temperature *T_p_*, the width of the damping peak (*ΔT*), which is defined as the spacing of the two temperatures at the half height of the damping peak (*tanδ*)_max_, is also an important parameter to characterize the damping capacity. The (*tanδ*)_max_ represents the maximum damping capacity, the *ΔT* determines the damping operating temperature range, and the *T_p_* permits the upper temperature limit for applications. According to the Debye expression for a single relaxation process, the theoretical width *ΔT_th_* of the damping peak can be expressed by *ΔT_th_* = 2.63*k_B_T_p_* ^2^/*H*.^[^ ^37^ ^]^ Thus, the *ΔT_th_* values of S3‐HT2, S3‐1.0O, and S3‐1.8O alloys at 1 Hz were calculated as 48, 52, and 53 K, respectively, which are much lower than the experimental *ΔT_ex_* values (146, 139, and 133 K, respectively). This phenomenon indicates that there exist several additional peaks in the damping curves of these three alloys, which might be related to multiple damping mechanisms regulated by the relaxation of interstitial O atoms in BCC lattice consisting of Zr, Ti, Hf, and Nb, and/or the reorientation of substitutional atoms.^[^ ^35^, ^36^, ^37^ ^]^

## 3 Discussion

### 3.1 Damping Mechanisms

From the above microstructural characterization (Figures 3 and 4), it can be observed that there exist diverse CSROs, which contain not only simple clusters composed of interstitial O atoms and separate substitutional atoms (Zr, Ti, Hf, and Nb), i.e., Zr‐O‐, Hf‐O‐, Ti‐O‐, and Nb‐O‐rich clusters, but also complex clusters, such as Zr‐Ti‐O‐, Hf‐Zr‐O‐, and Nb‐Zr‐Ti‐O‐rich clusters in O‐containing Zr_8_Hf_4_Ti_3_Nb_3_ (S3‐HT2) alloy. In fact, the stress‐induced reorientation of interstitial (I) atoms in the neighbors consisting of different substitutional (S) solutes could give rise to various Snoek‐type S‐I relaxation processes, corresponding to diverse O‐rich clusters.^[^ ^35^, ^56^ ^]^

**Figure 7:**
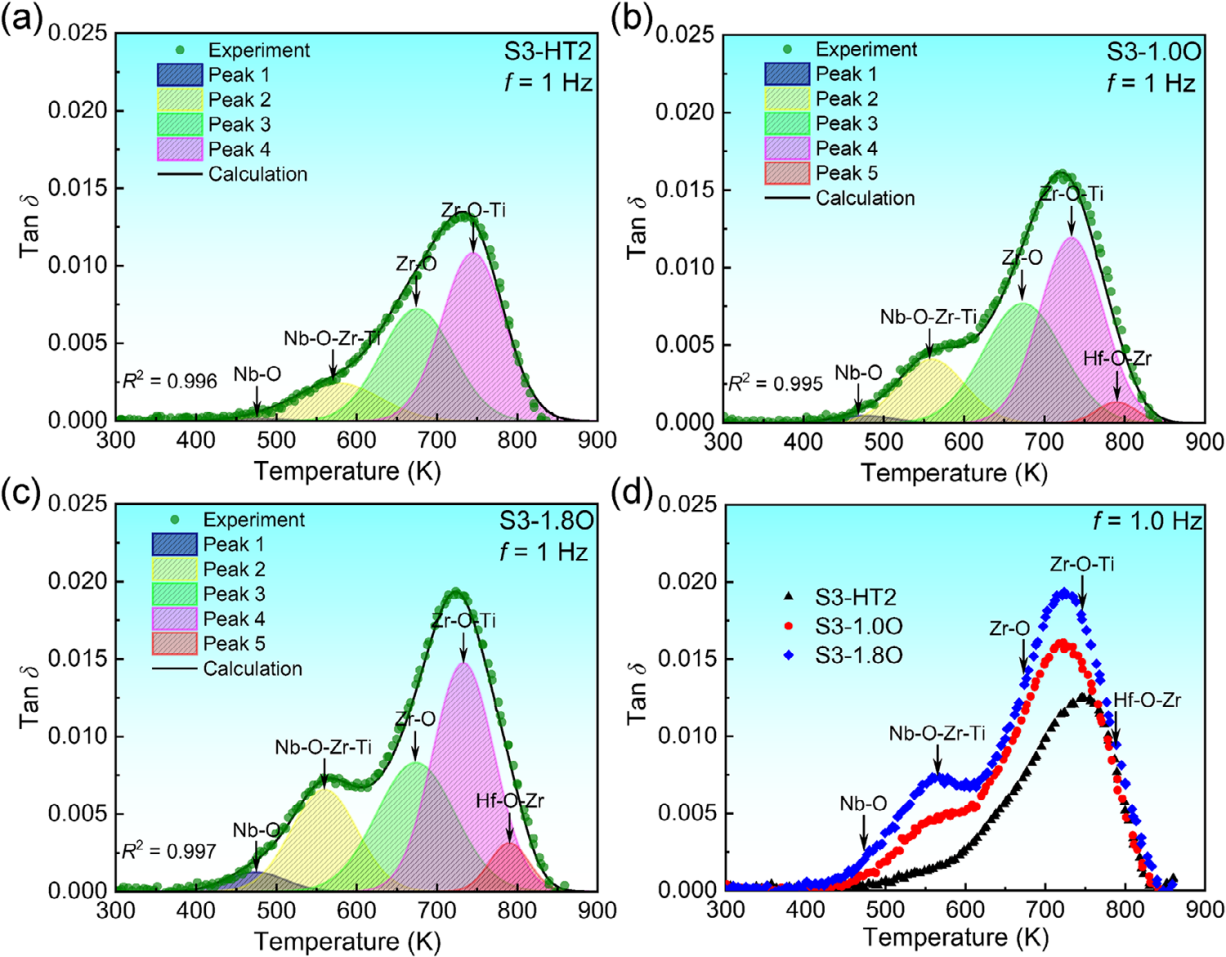
Damping mechanisms of the O‐free and O‐added S3‐HT2 alloys. Decomposition of damping peaks to analyze the damping capacity (tan*δ*) of a) S3‐HT2, b) S3‐1.0O, and c) S3‐1.8O alloys at 1 Hz, where the green dots represent the experimental results, while the black curves are the fitting sum of separated sub‐peaks that are labeled by shadows and curves with different colors. For comparison, the damping curves of these three alloys were plotted together in d), showing the absence of Peak 5 in O‐free alloy.

It is found that the damping peaks of the Zr_8_Hf_4_Ti_3_Nb_3_ alloys with and without the O addition exhibit an asymmetric broadening, which indicates that the damping behavior of these alloys involves multiple damping mechanisms.^[^ ^35^ ^]^ In order to investigate the damping mechanisms, the damping curves of these alloys at *f* = 1 Hz are separated into several sub‐peaks using the peak‐fitting module, as shown in **Figure** **7**, in which the higher correlation index of *R* ^2^ > 0.99 implies a more reliable fitting. The primary peak in the damping curve of O‐containing alloys can be decomposed into five sub‐peaks (Figure 7b,c), while that of the O‐free S3‐HT2 alloy consists of four sub‐peaks (Figure 7a). The damping capacity (*tanδ*)_max_ and corresponding peak temperature *T_p_* of each sub‐peak are listed in Table S3 (Supporting Information). It should be pointed out that the appearance of these individual peaks arises from the separate thermally‐activated S‐I relaxation processes due to the interactions between interstitial and substitutional atoms,^[^ ^35^, ^56^ ^]^ where the thermal activated temperatures for different S‐I processes are listed in Table S4 (Supporting Information).^[^ ^56^ ^]^ In particular, for S3‐1.8O alloy (Figure 7c), the first sub‐peak (Peak 1) with the lowest peak temperature of *T_p_*_1_ = 474 K is attributed to the stress‐induced reorientation of interstitial O atoms around Nb atoms, i.e., the Nb‐O relaxation process, corresponding to the Nb‐O clusters, since its peak temperature is comparable to the thermal activated temperature (380–476 K) for Nb‐O relaxation process (Table S4, Supporting Information). For the second sub‐peak (Peak 2), its peak temperature (*T_p_*_2_ = 560 K) is located exactly between the thermal activated temperature for the Nb‐O process and that (683–700 K) for Zr‐O. It has been demonstrated that the relaxation process involving the orientation of interstitial atoms in the neighbors of multiple substitutional solutes, i.e., the S‐I‐S′ process, indeed occurs at a higher temperature than that arising from the single S‐I process.^[^ ^56^ ^]^ Therefore, the Peak 2 derives from the stress‐induced switches of O atoms around Nb, Zr, and Ti atoms, i.e., the Nb‐O‐Zr‐Ti process, corresponding to the Nb‐Zr‐Ti‐O‐rich clusters. The third sub‐peak (Peak 3) with a peak temperature of *T_p_*_3_ = 674 K is dominated by the Zr‐O relaxation process with the thermal activated temperature (683–700 K), corresponding to the Zr‐O‐rich clusters. Analogously, the Peak 4 is regarded as the Zr‐O‐Ti relaxation, corresponding to the Zr‐Ti‐O‐rich clusters, because it possesses the highest damping capacity ((*tanδ*)_max_ = 0.015) due to its relatively‐high content of Zr and Ti. The last sub‐peak (Peak 5) with the highest peak temperature *T_p_*_5_ = 790 K is attributed to the stress‐induced jumping of interstitial O atoms in the neighbors of Hf and Zr atoms, i.e., the Hf‐O‐Zr relaxation process, corresponding to the Hf‐Zr‐O‐rich cluster. It is noted that the thermal activated temperature for Hf‐O‐Zr relaxation process is also higher than that (≈753 K) of the simple Hf‐O process. Similar phenomena occur in both O‐free and 1.0 at% O‐added alloys, except the absence of the Peak 5 in O‐free alloy due to the relatively‐lower O content, as shown in Figure 7d. Therefore, the multiple separated O‐rich clusters lead to different Snoek‐type damping mechanisms, which certainly broadens the damping peaks in these alloys.

The first‐principles density functional theory (DFT) calculations were conducted to investigate the energetics and electronic structures of S3‐HT2 alloys with and without the O addition (see details in “DFT calculations” section of Supporting Information). It is found that the O solution energies and formation energies of S3‐HT2 alloy with the O atom occupying three distinct octahedral interstices (Zr_4_Ti_2_‐O (Site 1), Zr_4_Ti_1_Hf_1_‐O (Site 2), Zr_4_Ti_1_Nb_1_‐O (Site 3) in Figures S7, S8, Supporting Information) are negative, as seen in Table S5 (Supporting Information), indicating that the BCC‐β structure is stable. This stability ensures the thermodynamic feasibility for forming diverse O‐rich clusters. Notably, the Zr_4_Ti_2_‐O site, having 4 Zr atoms and 2 Ti atoms as the nearest neighbor, exhibits the lowest O solution energy (*E*_O‐solu_ = −8.682 eV) and formation energy (*E*_f_ = −1.819 kJ mol^−1^), making it the most stable configuration (Table S5, Supporting Information). In contrast, when Hf or Nb atom substitutes for the Ti, both the O solution energy and formation energy tend to increase, which suggests that O atoms preferentially occupy the octahedral interstices adjacent to Zr‐ and Ti‐rich regions, thereby facilitating the formation of Zr‐Ti‐O‐rich clusters. This observation aligns with the microstructural characterization presented in Figures 3 and 4. Furthermore, it reinforces the finding that the Peak 4, attributed to Zr‐O‐Ti relaxation, possesses the highest damping capacity, with a maximum value of (*tanδ*)_max_ = 0.015 (Figure 7c).

**Figure 8:**
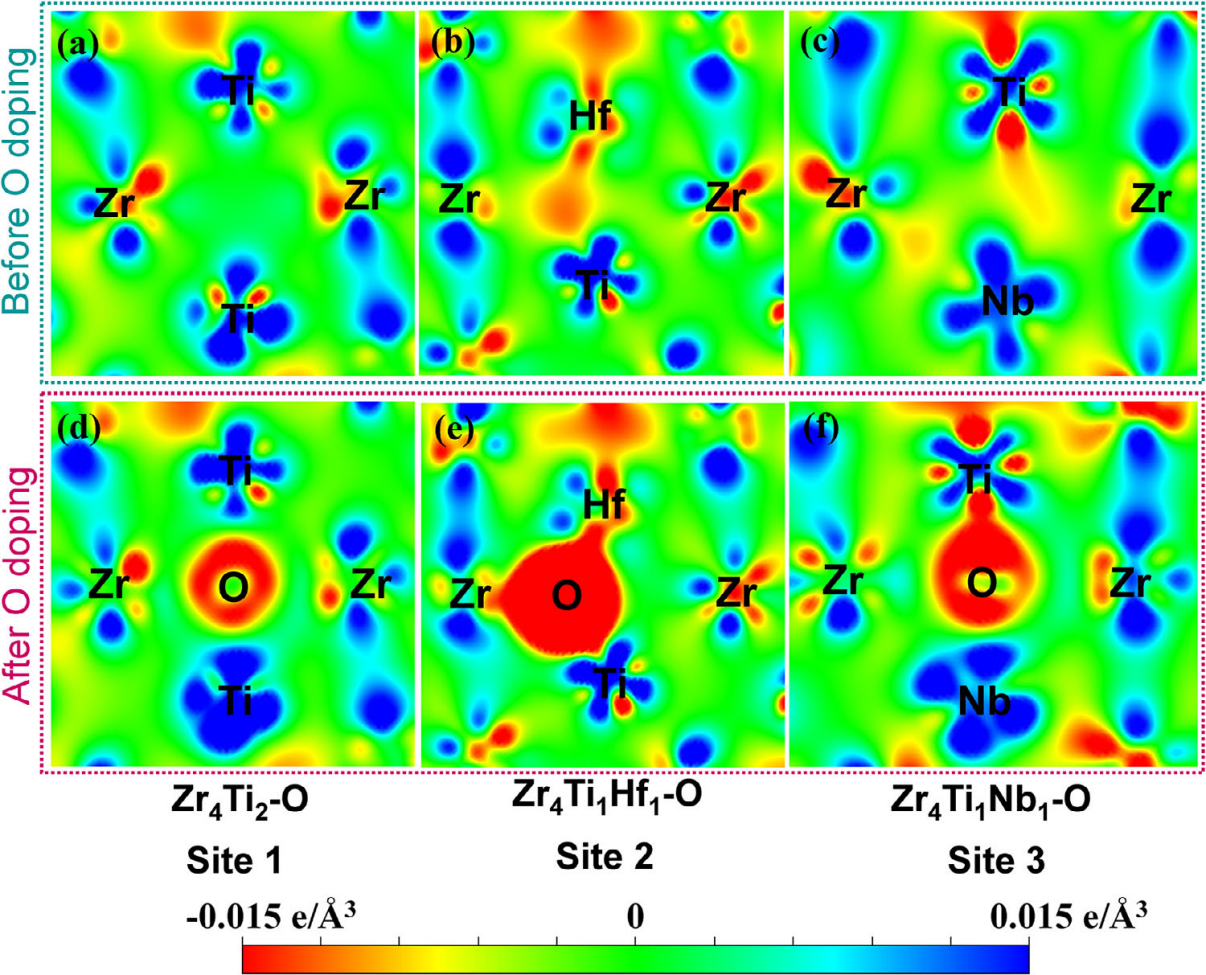
Differential charge density distributions on {110} surfaces in S3‐HT2 alloy with O atom occupying octahedral interstices of a,d) Zr_4_Ti_2_‐O (Site 1), b,e) Zr_4_Ti_1_Hf_1_‐O (Site 2), and c,f) Zr_4_Ti_1_Nb_1_‐O (Site 3) before and after O‐doping. The red and blue colors represent the charge accumulation and depletion, respectively.

Moreover, the bonding properties between O and diverse nearest‐neighbor metal atoms are analyzed through the Bader charge analysis and differential charge density distributions (**Figure** **8**; and Table S6, Supporting Information). After the O‐doping, Ti and Zr atoms lose a greater number of electrons, while the O atom gains electrons in Zr_4_Ti_2_‐O site. This increased charge transfer between O and Zr or Ti atoms is expected to convert the original metal‐metal bond to ionic‐like metal‐oxygen bond, thereby enhancing the atomic cohesion. In contrast, both Hf and O gain electrons in Zr_4_Ti_1_Hf_1_‐O site, indicating the covalent bonding between Hf‐O. While the Nb atom gains fewer electrons after the O‐doping in Zr_4_Ti_1_Nb_1_‐O site. It indicates that the ionic‐like bond between Nb‐O is weaker than those between Zr‐O and Ti‐O, and significantly weaker than the covalent‐like bond between Hf‐O, which is harder to break. It is observed that the separate thermally‐activated S‐I relaxation processes originate from stress‐induced reorientation of interstitial atoms around the neighboring metal atoms due to the interactions among these atoms, which is also characterized by the charge transfer. Consequently, the bond strength between O and metal atoms is closely related to the thermal activated temperatures associated with different S‐I processes. Thus, the strongest Hf‐O bond corresponds to the highest thermal activated temperature (*T_p_*_5_ = 790 K) for the Hf‐O‐Zr relaxation. And the weakest Nb‐O bond results in the lowest thermal activated temperature (*T_p1_* = 474 K) for the Nb‐O process.

It was reported that the Snoek damping capacity (*tanδ*)_max_ follows the linear relationship with the constituent concentration in a specific alloy system.^[^ ^35^, ^57^ ^]^ Thus, the Snoek damping capacity (*tanδ*)_max_ of 1.8 at% O‐added Zr_8_Hf_4_Ti_3_Nb_3_ alloy is higher than those of both O‐free and 1.0 at% O‐added alloys. Intriguingly, the atomic position switches of interstitials in the matrix could be impeded by the large atomic‐scale stress fields in MPEAs due to their high configuration entropy and severe lattice distortion.^[^ ^2^, ^3^ ^]^ This effect gives rise to a higher activation temperature and barrier energy for the reorientation of interstitial atoms, which finally results in a high damping capability at high temperatures.^[^ ^26^, ^58^ ^]^ Moreover, these alloys exhibit a significantly broader damping curve with a relatively higher peak value due to the occurrence of multiple separate O‐rich clusters induced by the co‐alloying of multi‐principal elements, which implies a prominent damping capacity over a wide temperature range (550–800 K).

### 3.2 Strengthening Mechanisms of O‐Free and O‐Added MPEAs

Interestingly, the addition of interstitial O can improve both the strength and ductility, as evidenced by the fact that the S3‐1.8O alloy exhibits the higher yield strength (*σ*_YS_ = 1000 MPa) and large ductility (*ε* = 15.1%), compared to the O‐free S3‐HT2 alloy (*σ*_YS_ = 704 MPa and *ε*  = 9.2%). Theoretically, the strength of the O‐free and O‐added MPEAs originates from collective contributions of various strengthening mechanisms, i.e., the substitutional solid‐solution strengthening (*Δσ*_ss_) from solute metal elements, interstitial solid solution strengthening (*Δσ*_is_) induced by the O addition, and grain boundary strengthening (*Δσ*_gb_) due to the grain refinement.^[^ ^38^ ^]^ Here, the precipitation strengthening is neglected since the amount of the secondary precipitates is very small. Therefore, the yield strength (*σ*_YS_) can be expressed with the following Equation (2):

(2) $$\sigma_{\mathrm{YS}}=\Delta \sigma_{\mathrm{ss}}+\Delta \sigma_{\mathrm{gb}}+\Delta \sigma_{\mathrm{is}}$$

The strength increment *Δσ*_ss_ from the substitutional solute elements was calculated by the Curtin's solid solution strengthening model based on the solute‐dislocation interaction energy, which has been proven in the Zr‐containing BCC‐based MPEAs.^[^ ^59^, ^60^, ^61^, ^62^ ^]^ Here, the *Δσ*_ss_ depends on the zero‐shear yield stress (*τ*_y0_) and the energy barrier (*ΔE*_b_) for thermally activated flow, being expressed with Equations (3)–(5):^[^ ^59^, ^60^ ^]^

(3) $$\Delta \;\sigma_{\mathrm{ss}}=M\tau_{y0}\left[1-{\left(\frac{k_{B}T}{\Delta E_{b}}\ln\frac{{\dot{\in }}_{0}}{\dot{\in }}\right)}^{\frac{2}{3}}\right]$$

(4) $$\tau_{y0}=A_{\tau }\;\alpha^{-\frac{1}{3}}G{\left(\frac{1+v}{1-v}\right)}^{\frac{4}{3}}{\left(\frac{\sum_{n}c_{n}\Delta V_{n}^{2}}{b^{6}}\right)}^{\frac{2}{3}}$$

(5) $$\Delta E_{b}=A_{E}\;\alpha^{\frac{1}{3}}Gb^{3}{\left(\frac{1+v}{1-v}\right)}^{\frac{2}{3}}{\left(\frac{\sum_{n}c_{n}\Delta V_{n}^{2}}{b^{6}}\right)}^{\frac{1}{3}}$$

where *M* = 2.73 is the Taylor factor for BCC structure,^[^ ^38^ ^]^ and *T* = 298 K is the thermodynamic temperature. ${\dot{\varepsilon }}_{0}$= 10^−4^ s^−1^ is the reference strain rate, while $\dot{\varepsilon }$ = 10^−3^ s^−1^ is finite strain rate.^[^ ^59^ ^]^ The prefactor coefficients of (*A_τ,_ A_E_*) = (0.04, 2.00) for BCC‐based alloys were obtained in light of the reduced elasticity theory.^[^ ^60^ ^]^ *α* = 0.0833 for the BCC structure is related to the edge dislocation line tension.^[^ ^60^ ^]^ *G* is the shear modulus, which is calculated with the formula of *G* = *E*/2(1+*ν*) according to the measured Young's modulus *E* = 49–69 GPa (see Table S1, Supporting Information) from the tensile test and the Poisson's ratio of *ν* = 0.39.^[^ ^38^ ^]^ *b* = $\frac{\sqrt{3}}{2}$ *a* is the Burgers vector.^[^ ^38^ ^]^ *c_n_* and *ΔV_n_* are the atomic concentration and misfit volume of the solute element *n* in the solid solution, respectively. The *ΔV_n_* is calculated with the formula of *ΔV_n_* = *V_n_* − *V*_alloy_, in which *V_n_* is the apparent atomic volume of pure element *n* in BCC structure, being 23.02 Å^3^ for Zr, 22.528 Å^3^ for Hf, 17.387 Å^3^ for Ti, and 17.952 Å^3^ for Nb,^[^ ^59^ ^]^ and *V*_alloy_ = *a* ^3^/2 is the atomic volume of BCC alloys.^[^ ^59^ ^]^ The difference in apparent volumes among these elements can produce a large misfit volume *ΔV_n_*, inducing a remarkable solid‐solution strengthening effect. Thus, the *Δσ*_ss_ is estimated to be 480 MPa for S1‐T alloy, 505 MPa for S2‐HT1 alloy, and 630 MPa for S3‐HT2 alloy.

The contribution (*Δσ*_gb_) from the grain boundary strengthening can be addressed by the Hall‐Petch relationship, as expressed with **Equation (6)**:^[^ ^63^ ^]^

(6) $$\Delta \sigma_{gb}=\sigma_{0}+K_{\mathrm{HP}}D^{-\frac{1}{2}}$$

where *σ*_0_ is the lattice friction stress of BCC‐Zr alloys, commonly taken as ≈26 MPa.^[^ ^62^ ^]^ *K*_HP_ = 270 MPa µm^−1/2^ is the Hall‐Petch coefficient, which has been used for HfNbTaZrTi MPEA.^[^ ^59^, ^61^ ^]^ Since the grain size (*D*) of these current O‐free MPEAs is 100–130 µm, the strength increment is calculated to be *Δσ*_gb_ = 50–53 MPa. Thus, the estimated yield strength values for these three O‐free alloys can be calculated with the formula *σ*_YS_ = *Δσ*_ss_ + *Δσ*_gb_, being 529 MPa for S1‐T alloy, 558 MPa for S2‐HT1 alloy, and 681 MPa for S3‐HT2 alloy, which is consistent with the experimentally‐measured values (554–704 MPa).

For the O‐added MPEAs, when O atoms reside in the octahedral interstitial sites of BCC lattice, it could produce an asymmetric local strain, i.e., the tetragonal distortion, which effectively hinders the motion of dislocations.^[^ ^64^ ^]^ Therefore, the interstitial solid‐solution strengthening contribution from this tetragonal distortion is much larger than that from the symmetric distortion induced by substitutional solute elements.^[^ ^65^ ^]^ Thus, the strength increment *Δσ*_is_ caused by the interstitial O atoms could be expressed by the Fleischer's equation (Equation (7)):^[^ ^66^ ^]^

(7) $$\Delta \sigma_{is}=\frac{1}{3}\;G\Delta \varepsilon c^{1/2}$$

where *Δε* = 0.10 is the difference between the longitudinal and transverse strain of the tetragonal distortion when the O atoms occupy the interstitial sites in BCC lattice.^[^ ^25^, ^62^ ^]^ *c* is the atomic concentration of interstitial elements in the solid solution. Since the concentration of N is far below that of O, only the O content was considered. According to the measured O content, *Δσ*_is_ is calculated as 23 MPa for the S3‐HT2 alloy and 237 MPa for the S3‐1.8O alloy. For comparison, the strength contributions from each individual mechanism in S3‐HT2 and S3‐1.8O alloys are summarized in Figure 5c. To combine the intrinsic strength increment, the resultant yield strength values of S3‐HT2 and S3‐1.8O are ≈705 and ≈995 MPa, respectively, being well consistent with the measured values (704 and 1000 MPa, respectively). It is found that the contribution of O in enhancing the strength cannot be neglected in these designed O‐added MPEAs, although the solid‐solution strengthening (*Δσ*_ss_) from substitutional solute elements is dominant.

## 4 Conclusion

In conclusion, we develop a series of oxygen‐enhanced Zr‐Hf‐Ti‐Nb MPEAs with high strength and outstanding damping capacity through tailoring the BCC structural stability by an elaborate composition regulation with the cluster formula of [Ti‐(Zr,Hf,Ti)_14_]Nb_3_. The designed Zr_14_TiNb_3_ and Zr_8_Hf_6_TiNb_3_ alloys possess low BCC‐β structural stability because a minor amount (0.2–0.5 at%) of oxygen additions can induce the precipitation of α'' and ω. While the Zr_8_Hf_4_Ti_3_Nb_3_ alloy has a much higher BCC‐β stability, as evidenced by the fact that only few α'' and ω precipitates appear in 1.8 at% oxygen‐added alloy. This alloy exhibits an optimal mechanical property with a higher yield strength (*σ*_YS_ = 1000 MPa) and larger ductility (*ε* = 15.1%), compared to the oxygen‐free alloy (*σ*_YS_ = 704 MPa and *ε* = 9.2%), which is ascribed to the solid strengthening contribution from both the substitutional and interstitial elements. Moreover, the Zr_8_Hf_4_Ti_3_Nb_3_ alloys with and without oxygen additions exhibit an excellent damping capacity due to their low Young's modulus (*E* < 70 GPa), as exemplified with a peak value of (*tanδ*)_max_ = 0.02 for 1.8 at% oxygen‐added alloy. Notably, the damping characteristics are prominent over a wide temperature range (550—800 K), which is controlled by diverse Snoek‐type relaxation processes induced by the stress‐induced reorientation of interstitial O atoms in the neighbors of substitutional solutes, corresponding to each separate O‐rich cluster.

## 5 Experimental Section

### Sample Preparation

Alloy ingots of Zr_14_TiNb_3_ (S1‐T), Zr_8_Hf_6_TiNb_3_ (S2‐HT1), and Zr_8_Hf_4_Ti_3_Nb_3_ (S3‐HT2), together with those doped by different oxygen contents (0.2–2 at%), were prepared by means of arc melting under an argon atmosphere, in which the purity of each raw metal was 99.99 wt. % and the introduction of O was realized by adding TiO_2_ powders. Each ingot with a weight of ≈100 g was remelted at least five times to ensure the chemical homogeneity. Then these ingots were cold‐rolled for multiple passes to achieve plates with a thickness of 1.5 mm. Finally, these plates were sealed in vacuum quartz glass tubes and homogenized at 1223 K for 0.5 h in a muffle furnace.

### Microstructural Characterization

Crystalline structures of these homogenized alloys were identified using a Bruker D8 XRD with a Cu‐*K*_α_ radiation (*λ* = 0.15406 nm) and a scanning speed of 2 °⋅min^−1^, in which an external standard method was applied to calculate the lattice constants of phases (*56*). Microstructures were examined by the Olympus OM, JEOL‐JSM‐IT800SHL scanning electron microscope (SEM), and JEOL‐JEM‐2100F field‐emission TEM. Alloy samples for OM and SEM observations were mechanically ground, polished, and then etched in a mixed solution of 5 mL HF + 15 mL HNO_3_ + 80 mL C_2_H_5_OH. TEM specimens were prepared by twin‐jet electron‐polishing in a mixed solution of 60 mL CH_3_OH + 34 mL CH_3_(CH_2_)_3_OH + 6 mL HClO_4_ at ≈243 K. EBSD samples were prepared by argon‐ion‐beam polishing. The chemical composition was analyzed using SHIMADZU EPMA with a Super‐X energy dispersive and XRF‐1800 X‐ray fluorescence spectrometer (XRF). Inert gas fusion technique was used to measure oxygen and nitrogen by the Eltra Elementrac O/N/H analyzer, in accord to the ASTM E 1019 standard. A probe aberration‐corrected FEI‐Titan‐Themis STEM with an iDPC technique was used to analyze the O interstitial positions in the BCC lattice. The O content in the homogenized alloys was measured using an ELTRA ELEMENTRAC Oxygen‐nitrogen‐hydrogen analyzer. APT characterizations were performed in a local electrode atom probe (CAMECA LEAP 5000 XR), where the needle‐shaped specimens were fabricated by lift‐outs and annular milled in a FEI Scios FIB/SEM and were analyzed at 70 K in voltage mode with a pulse repetition rate of 200 kHz, a pulse fraction of 20%, and an evaporation detection rate of 0.2% atom per pulse.^[^ ^68^ ^]^ The data analysis software AP Suite 6.1 was used to create the 3D reconstructions and to analyze the data.^[^ ^69^ ^]^

### Property Testing

Uniaxial tensile tests were executed on a UTM5504 material test system with a strain rate of 10^−3^ s^−1^ at room temperature, in which a strain gage was applied during the elastic deformation to precisely measure the Young's modulus and three tensile plates for each alloy were prepared with a gauge size of 26.0 × 5.0 × 1.5 mm (length × width × thickness). The damping capacity of the designed alloys were tested by multifunction internal friction apparatus (MFP‐1000) at the frequency of *f* = 0.5, 1.0, 2.0, and 4.0 Hz over a temperature range of 300–900 K with a heating rate of 2 K min^−1^. The beam‐shaped samples with a dimension of 1 mm × 1.5 mm × 40 mm were polished before testing, and the forced vibration mode was applied with a maximum strain amplitude of 2 × 10^−5^ during testing.

### Theoretical Calculations

The first‐principles DFT calculations were conducted using the plane‐wave (PW) and projector‐augmented wave (PAW) method as implemented in the Vienna ab initio Simulation Package (VASP).^[^ ^70^, ^71^ ^]^ The electronic exchange correlation potential was described using the generalized‐gradient approximation (GGA) parametrized by Perdew, Burke, and Ernzerhof (PBE).^[^ ^72^ ^]^ The Ti‐3p^6^3d^2^4s^2^, Zr‐4s^2^4p^6^5s^2^4d^2^, Hf‐5p^6^6s^2^5d^2^, Nb‐4p^6^4d^4^5s^1^, and O‐2s^2^2p^4^ are treated as the valence states. The kinetic energy cutoff for the plane‐wave basis was set to 500 eV. Ions and lattice parameters of the structural model were permitted to fully relax, achieving a Hellmann‐Feynman force tolerance of 0.02 eV Å^−1^ and an energy tolerance of 1 × 10^−6^ eV during the geometrical optimizations. The k‐point mesh was set as the 7 × 7 × 2 Monkhorst‐Pack grid with a density of 0.02 Å^−1^ for the unit cells.

The cluster structural unit of [Ti‐Zr_8_Hf_4_Ti_2_](Nb_3_) for S3‐HT2 alloy containing 18 atoms was constructed based on the CSROs in solid solutions and has been demonstrated to be the most stable structure due to the lowest system energy.^[^ ^29^, ^73^, ^74^, ^75^ ^]^ Here, it was extended to a super cluster unit with 72 atoms along the original *z* axis, as presented in Figure S7 (Supporting Information). Then it was embedded into the current first‐principles calculations as the model input. To investigate the effect of interstitial oxygen on the system energy of this alloy, one oxygen atom was inserted into an octahedral interstice, corresponding to an O concentration of 1.4 at%. Thus, the O solution energy (*E*_O‐solu_) was calculated with Equation (8):

(8) $$E_{O-\mathrm{solu}}=E_{\mathrm{alloy}+O}\;-E_{\mathrm{alloy}}-E_{O}$$

where, *E*_alloy+O_ is the total energy of the structural model with an O atom inserted in matrix after relaxation, *E*_alloy_ is the energy of the model without O atom, and *E*_O_ is the energy of an O atom in a vacuum. And in order to evaluate the effect of O addition on the BCC‐β structural stability, the formation energies (*E*_f_), generally representing the required energy for the formation of an alloy at 0 K, were expressed with Equation (9):

(9) $$E_{f}=\left(E_{\mathrm{alloy}+O}-\sum E_{M}\right)/73$$

where *E*_M_ is the total energy per atom of the element M with a stable structure, in which the stable structures of M are HCP for Zr, Ti, and Hf, and BCC for Nb, respectively.

## Conflict of Interest

The authors declare no conflict of interest.

## Supporting information



Supporting Information

## Data Availability

The data that support the findings of this study are available from the corresponding author upon reasonable request.
