# Supplementary material for: Achieving High Damping Capacity in Oxygen‐Enhanced BCC Zr‐Hf‐Ti‐Nb Multi‐Principal‐Element Alloys with Low Young's Modulus
Source: Adv Sci (Weinh). 2025 Apr 29;12(25):2501068. doi: 10.1002/advs.202501068 (PMC12225015; doi:10.1002/advs.202501068)
Supplement: Supplementary file 1 — Supporting Information [file ADVS-12-2501068-s001.docx]

Supporting Information

Achieving high damping capacity in oxygen-enhanced BCC Zr-Hf-Ti-Nb multi-principal-element alloys with low Young’s modulus

Qing Wang,* Zhenhua Wang, Qixiang Zhang, Rui Wang, Tongmin Wang, Chaoli Ma, Ang Li,* Xiaodong Han, Junhua Luan, Zengbao Jiao,* P. K. Liaw

Qing Wang, Zhenhua Wang, Qixiang Zhang, Rui Wang, Tongmin Wang

School of Materials Science and Engineering, Key Laboratory of Materials Modification by Laser, Ion and Electron Beams (Ministry of Education), Dalian University of Technology, Dalian 116024, China
E-mail: [wangq@dlut.edu.cn](mailto:wangq@dlut.edu.cn) (Q. Wang).

Chaoli Ma

Tianmushan Laboratory, Yuhang District, Hangzhou 310023, China

Ang Li, Xiaodong Han

Beijing Key Laboratory of Microstructure and Property of Advanced Materials, Faculty of Materials and Manufacturing, Beijing University of Technology, Beijing, China

Junhua Luan

Inter-University 3D Atom Probe Tomography Unit, Center for Advanced Nuclear Safety and Sustainable Development, City University of Hong Kong, Hong Kong, China

Zengbao Jiao

Department of Mechanical Engineering, Research Institute for Advanced Manufacturing, The Hong Kong Polytechnic University, Hong Kong, China

P. K. Liaw

Department of Materials Science and Engineering, University of Tennessee, Knoxville, Tennessee 37996, USA

**Composition design via the cluster formula approach**

The cluster formula approach is based on the chemical short-range orders (CSROs) in solid solutions. And it consists of the nearest-neighbor cluster and the second-nearest-neighbor glue atoms, as expressed with the formula of [cluster](glue atoms)*_m_* (*m* being the number of glue atoms).^[S1-S3]^ Among them, the nearest-neighbor cluster is centered by the solute atom that has a strong interaction (characterized by a large negative enthalpy of mixing *ΔH*) with the base solvent atoms, and the glue atoms generally fill the space among clusters for balancing the atomic-packing density. Particularly, in the BCC structure, the cluster is the rhombi-dodecahedron with a coordination number (CN) of 14, and the glue atom number was ideally calculated as *m* = 1 or 3 with the guide of the Friedel oscillation theory for electron-structure stabilization. The value of *m* = 1 or 3 is determined by the fact that whether the strong BCC-stabilized elements (Mo, V, etc.) or the weak BCC-stabilized elements (Nb, Ta, etc.) were added.^[S3]^ In the Zr-Hf-Ti-Nb alloy series, Nb atoms would like to occupy the glue atom sites due to the positive enthalpies of mixing among Nb-Ti, Nb-Zr, and Nb-Hf, being *ΔH*_Nb-Ti_ = 2 kJ⋅mol^-1^, *ΔH*_Nb-Zr_ = 4 kJ⋅mol^-1^, and *ΔH*_Nb-Hf_ = kJ⋅mol^-1^, respectively.^[S4]^ While Ti, Zr, and Hf can occupy both the cluster center and cluster shell because they are in the same group, showing a nearly zero mixing enthalpy among them.^[S4]^ Moreover, it has been demonstrated that Nb is a relatively weaker BCC-stabilizer compared with those strong BCC-stabilizers (such as Mo).^[S3]^ Thus, the cluster formula with *m* = 3 was often used to characterize the BCC structural stability in Zr-Hf-Ti-Nb system.^[S2]^ Finally, the basic cluster formula can be expressed as [M-M_14_](Nb_3_), where M represents any element of Ti, Zr, and Hf or their combination.

In our previous works, we designed three series of alloys, the Zr-Nb series (N series), the Zr-Nb-Ti series (T series), and the Zr-Hf-Ti-Nb series (H series) via the cluster formula of [M-M_14_](M,Nb)_3_, to investigate the effect of alloying elements on the BCC structural stability.^[29]^ Firstly, the binary Zr-Nb (N series) is selected as the basic system due to that the BCC structural stability could be enhanced with increasing the Nb content. The single BCC-β structure appears in N3-Zr_15_Nb_3_ alloy combined with a minor amount of , while the HCP-α phase dominates in N1-Zr_17_Nb_1_ alloy and a large amount of  nanoparticles precipitate from the BCC-β matrix in N2-Zr_16_Nb_2_ alloy. However, it is noted that the metastable phases of α', α'', and , are always coexisted with the BCC-β matrix even when the Nb content is higher than the critical lower limit (~ 20 at. % Nb) for BCC stabilization.^[S5]^ Then, when an appropriate amount of Ti substitutes for Zr in the N3-Zr_15_Nb_3_ alloy, the BCC-β structural stability is indeed enhanced, as evidenced by the fact that the T1-Zr_14_Ti_1_Nb_3_ alloy possesses a single BCC-β structure without any  precipitation. However, the excessive Ti will deteriorate the BCC structural stability of alloys after the further substitution of Ti for Nb, because a large amount of the  nanoparticles appear in both T2-Zr_14_Ti_2_Nb_2_ and T3-Zr_14_Ti_3_Nb_1_ alloys. Finally, when the Hf element completely substitutes for Zr in the T1-Zr_14_Ti_1_Nb_3_ alloy, a large of amount of  nanoparticles appear in H1-Hf_14_Ti_1_Nb_3_ alloy, which indicates that the BCC-stabilized capacities of Nb and Ti elements in Hf-based alloys are weaker than those in Zr-based alloys. While the co-existence of Zr and Hf can reduce the amount of  nanoparticles in H2-Zr_8_Hf_6_Ti_1_Nb_3_ alloy. In addition, an appropriate amount of Ti substitution for Hf can further enhance the BCC stability, as evidenced by the H3-Zr_8_Hf_4_Ti_3_Nb_3_ alloy with a single BCC structure, in which the  nanoparticles disappear. Importantly, these alloys dominated by the BCC structure have a lower Young’s modulus, being *E* = 50 ~ 70 GPa. Otherwise, the modulus will be increased under the condition of a large amount of  precipitation. It is known that a lower *E* is necessary for high-damping alloys to eliminate noise and mechanical vibrations, since the ratio of loss modulus to storage modulus, *i.e.*, *tanδ*, represents the damping capacity under the condition that the internal friction is anelastic.^[35, 36]^ Therefore, we selected three alloys with higher BCC-β structural stability, Zr_14_Ti_1_Nb_3_ (T1), Zr_8_Hf_6_TiNb_3_ (H2), and Zr_8_Hf_4_Ti_3_Nb_3_ (H3), as the basic alloys to investigate the influence of O addition on the BCC stability, mechanical properties and damping capacities of alloys. Here, these three alloys were re-labeled as S1-T, S2-HT1, and S3-HT2, respectively, for a better identification.

**DFT calculations**

In order to elucidate the effect of octahedral interstices with different nearest-neighbor metal atoms occupied by the O atom on the BCC structural stability, three distinct octahedral interstice sites were selected, including Site 1 (Zr_4_Ti_2_-O, i.e., the nearest neighbor consists of 4 Zr atoms and 2 Ti atoms), Site 2 (Zr_4_Ti_1_Hf_1_-O), and Site 3 (Zr_4_Ti_1_Nb_1_-O), as shown in Figure S8. Table S5 presents the O solution energies and formation energies of S3-HT2 alloy with the O atom occupying these three specified sites. It is found that the O solution energies and formation energies for these three sites are negative, indicating that they are stable. This stability ensures the thermodynamic feasibility for forming diverse O-rich clusters. Notably, the Site 1, having 4 Zr atoms and 2 Ti atoms as the nearest neighbor, exhibits the lowest O solution energy (*E*_O-solu_ = -8.682 eV) and formation energy (*E*_f_ = -1.819 kJ⋅mol^-1^), making it the most stable configuration. In contrast, when Hf or Nb atom substitutes for the Ti at Site 2 or Site 3, both the O solution energy and formation energy tend to increase. This trend suggests that oxygen atoms preferentially occupy the octahedral interstices adjacent to Zr- and Ti-rich regions, thereby facilitating the formation of Zr-Ti-O-rich clusters. This observation aligns with the microstructural characterization presented in Figures 3 and 4. Furthermore, it reinforces the finding that the Peak 4, attributed to Zr-O-Ti relaxation, possesses the highest damping capacity, with a maximum value of (*tanδ*)_max_ = 0.015 (Figure 7c).

To further investigate the bonding properties between O atom and diverse nearest- neighbor metal atoms, local charge transfer between oxygen and metal atoms is analyzed through differential charge density distributions and the Bader charge analysis, where the latter can quantitatively characterize the charge transfer between atoms.^[S6]^ The variation of Bader charge (charge transfer) of O and metal atoms for the current three sites, both before and after O-doping, are listed in Table S6. Furthermore, the differential charge density distributions on the {110} surfaces of model before and after the O-doping are illustrated in Figure 8. It can be seen that after doping the O atom, Ti and Zr atoms lose a greater number of electrons, while the O atom gains electrons in Site 1 (Zr_4_Ti_2_-O). This increased charge transfer between O and Zr or Ti atoms is expected to convert the original metal-metal bond to ionic-like metal-oxygen bond, thereby enhancing the atomic cohesion. In Site 2 (Zr_4_Ti_1_Hf_1_-O), the Bader charge of Hf atom decreases from the original 1.16 *e*^-^ (without O-doping) to -1.89 *e*^-^ with a difference of 3.05 *e*^-^, indicating a significant charge transfer, which is greater than those of Zr (0.33 *e*^-^) and Ti (1.0 ~ 1.54 *e*^-^) atoms. Notably, compared with the Ti and Zr atoms that lose electrons, both Hf and O gain electrons in Site 2, indicating the covalent bonding between Hf and O. In Site 3 (Zr_4_Ti_1_Nb_1_-O), both Nb and O atoms gain electrons, but the Nb atom receive fewer electrons, as evidenced by the Bader charge of the Nb decreasing from 0.89 *e*^-^ to 0.13 *e*^-^. It indicates that the ionic-like bond between Nb-O is weaker than the bonds between Zr-O and Ti-O, and significantly weaker than the covalent-like bond between Hf-O, which that is harder to break. It is observed that the separate thermally-activated S-I relaxation processes are a result of stress-induced reorientation of interstitial atoms around the neighboring metal atoms due to the interactions among these atoms, which is also characterized by the charge transfer. Consequently, the bond strength between oxygen and metal atoms is closely related to the thermal activated temperatures associated with different S-I processes. And the strongest Hf-O bond corresponds to the highest thermal activated temperature (*T_p_*_5_ = 790 K) for Hf-O-Zr relaxation. In constrast, the weakest Nb-O bond results in the lowest thermal activated temperature (*T_p1_* = 474 K) for the Nb-O process.


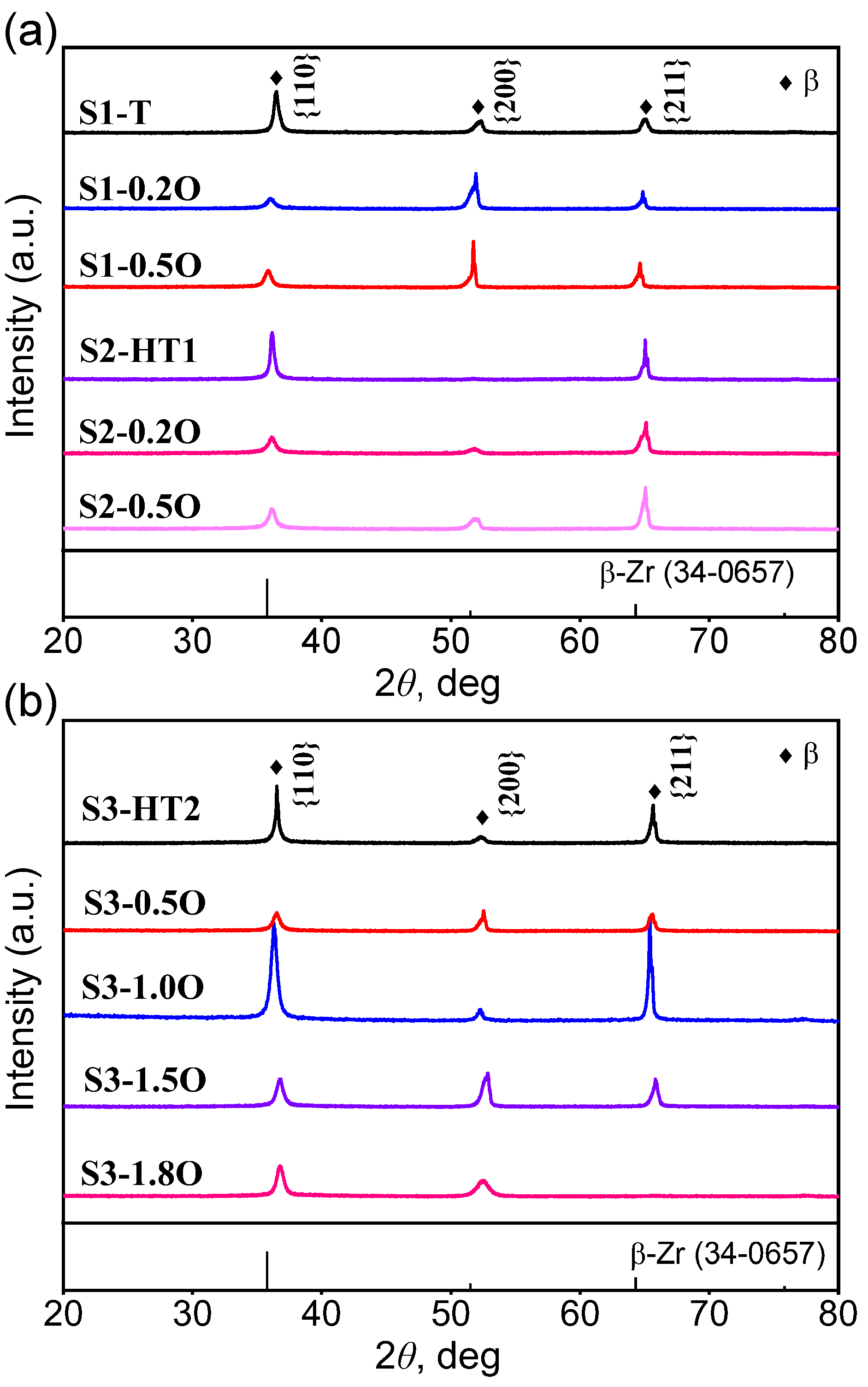


**Figure S1.** XRD patterns of the designed alloys containing different O contents. a): S1-T and S2-HT1 alloys with 0 ~ 0.5 at. % O, and b): S3-HT2 alloys with 0 ~ 1.8 at. % O.


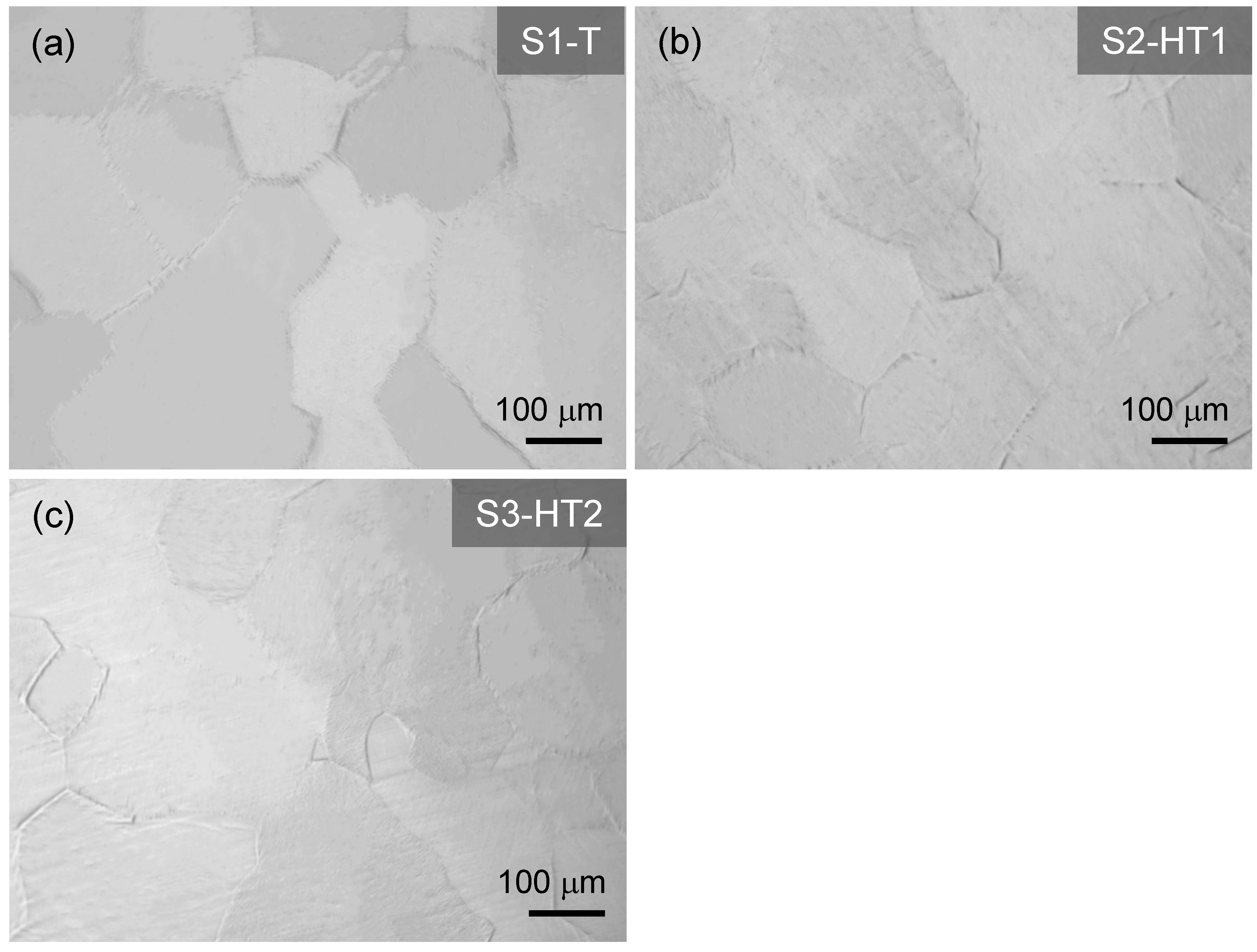


**Figure S2.** OM images of the oxygen-free alloys. a): S1-T, b) S2-HT1, and c): S3-HT2.


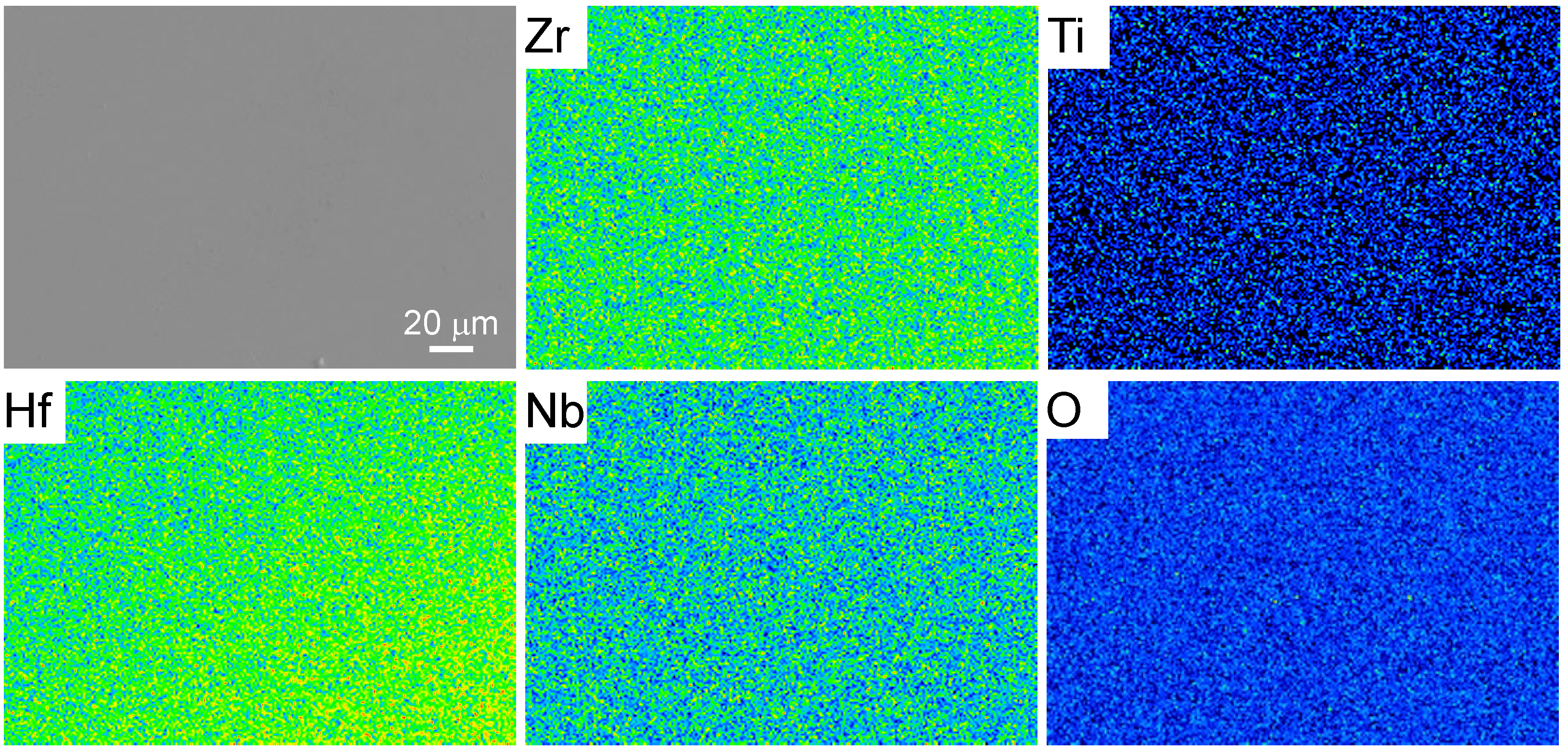


**Figure S3.** Elemental distributions in S3-1.8O alloy mapped with EPMA.


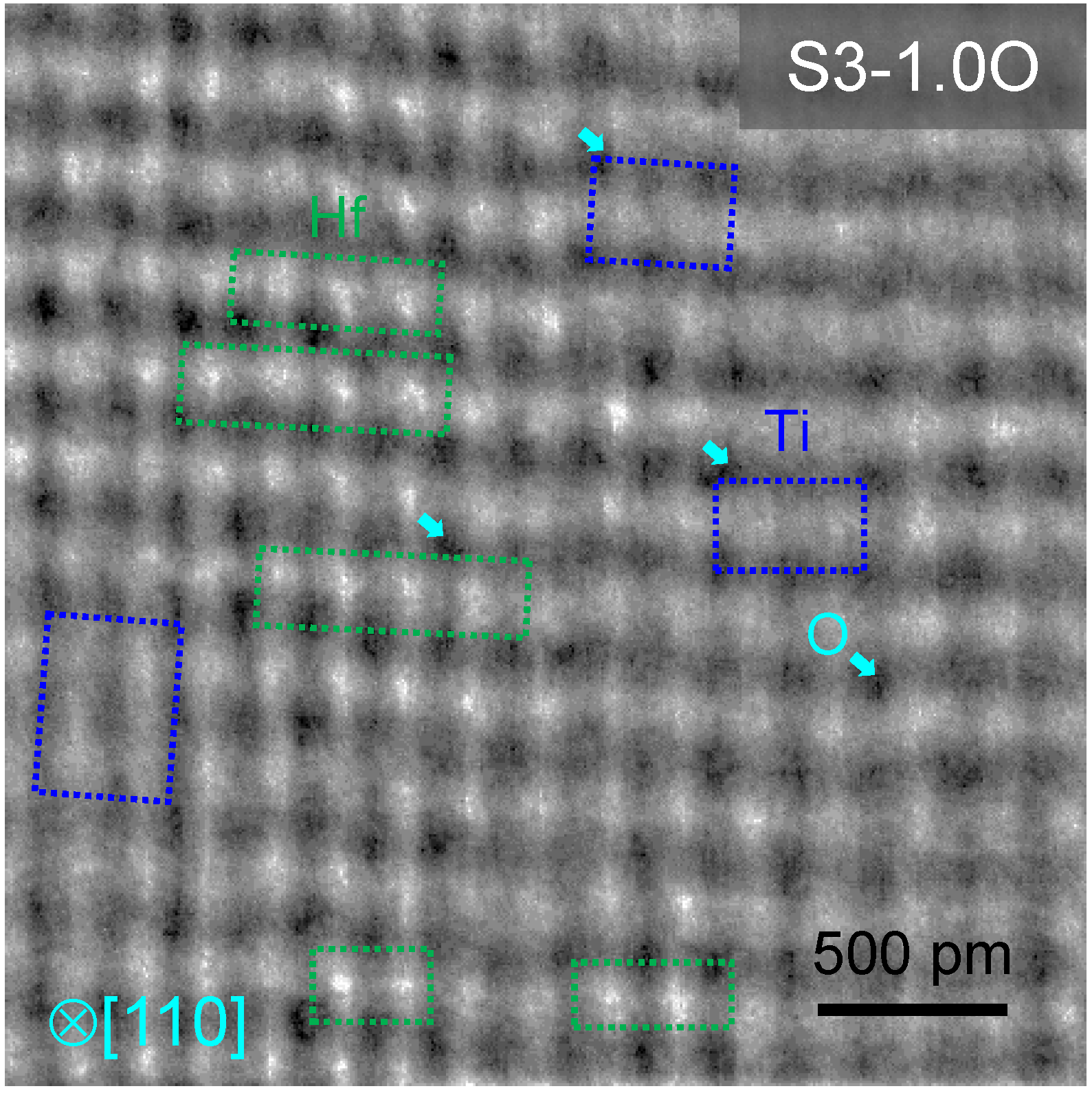


**Figure S4.** The iDPC-STEM image of in S3-1.0O alloy along the [110] direction.


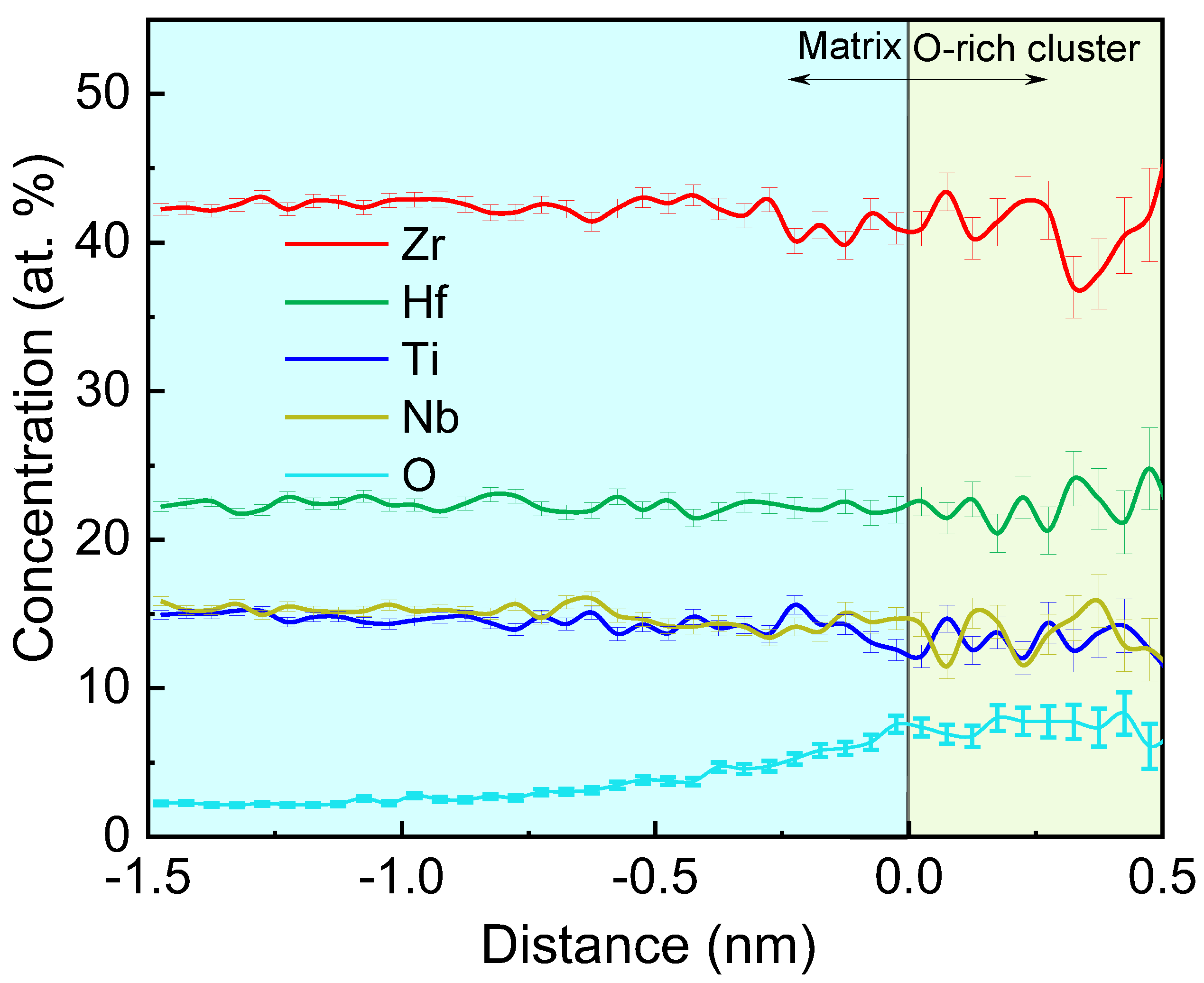


**Figure S5.** Proximity histogram as a function of distance to the concentration isosurface of 3.5 at. % O base on the APT characterization of the S3-1.8O alloy, showing the elemental distribution in O-rich clusters.


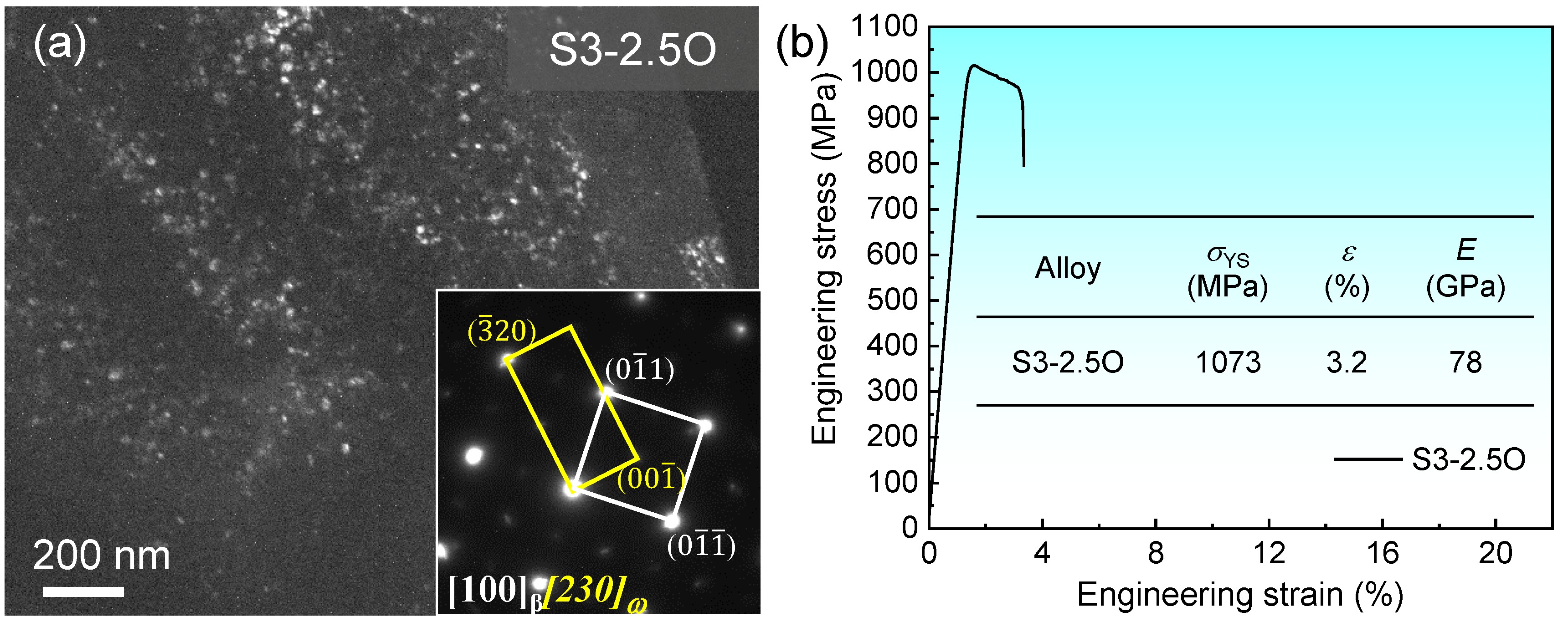


**Figure S6.** a) DF-TEM image and corresponding SAED pattern along the [100]_β_ direction of S3-2.5O alloy, showing a large amount of ω nanoparticles precipitate from the BCC-β matrix; b) Tensile engineering stress-strain curve of S3-2.5O alloy, where the yield strength (*σ*_YS_), elongation to fracture (*ε*), and Young’s modulus (*E*) are also listed.


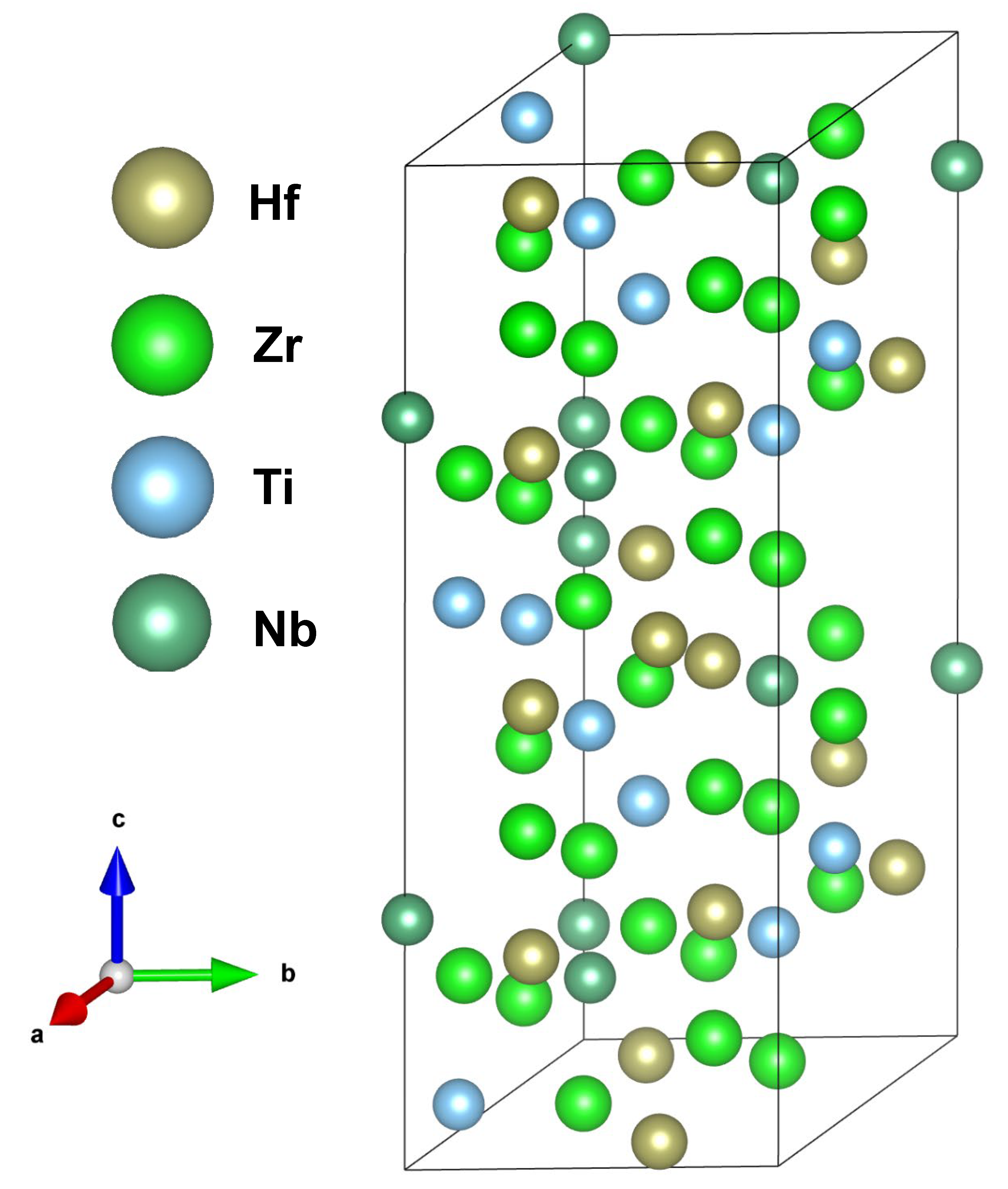


**Figure S7**. Cluster structural model containing 72 atoms, which was embedded into the first-principles calculations as the model input.


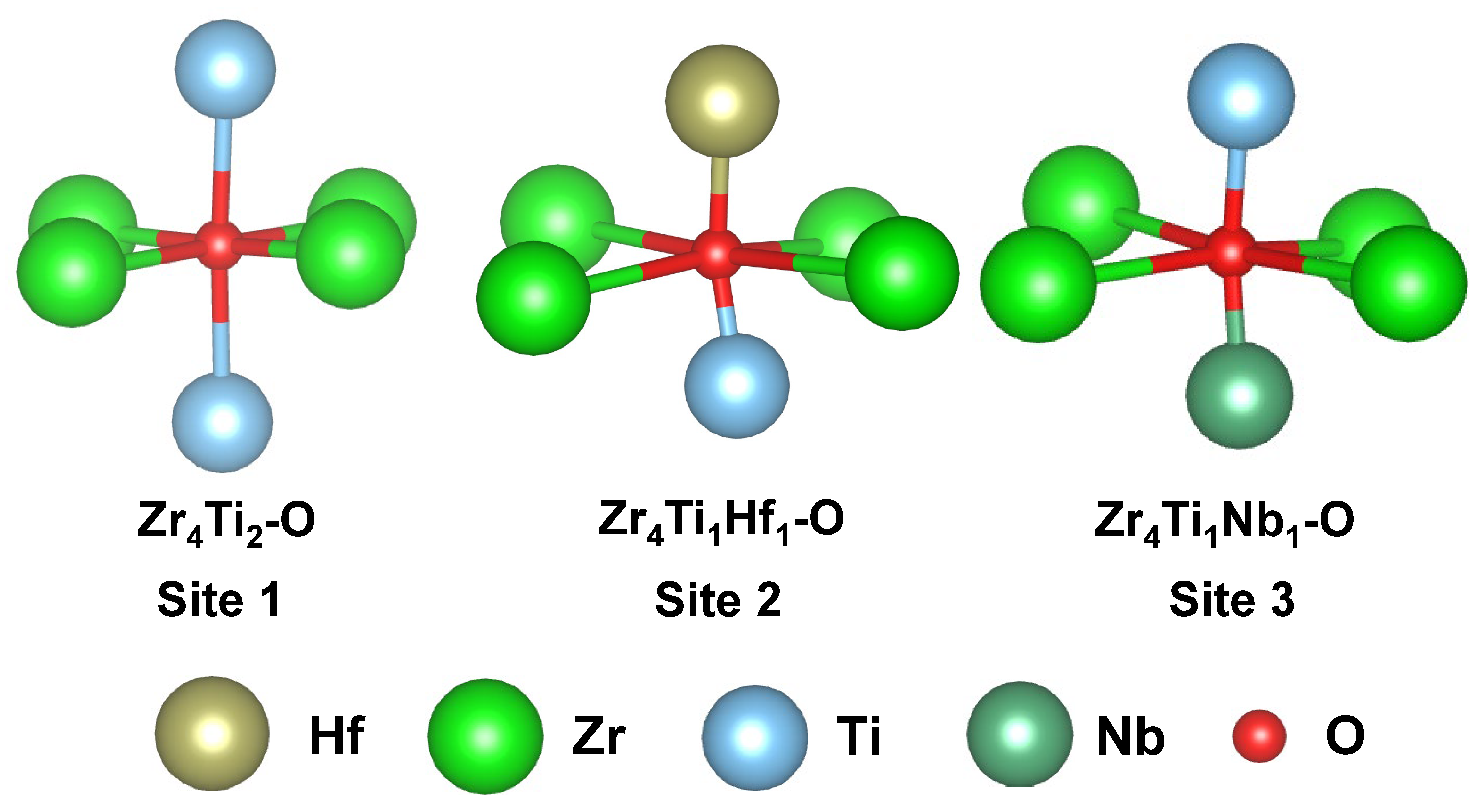


**Figure S8.** Structures for the S3-HT2 alloy with the O atom occupying the octahedral interstices of Site 1 (Zr_4_Ti_2_-O), Site 2 (Zr_4_Ti_1_Hf_1_-O), and Site 3 (Zr_4_Ti_1_Nb_1_-O), respectively.

**Table S1.** Data summary for the designed MPEAs, including the cluster formulas, the compositions in atomic percent (at. %), phase constitutions, lattice constant (*a*) of the matrix, yield strength (*σ*_YS_), ultimate tensile strength (*σ*_UTS_), elongation to fracture (*ε*), and Young’s modulus (*E*).

| Alloys | Cluster formulas | Compositions [at. %] | Phase constitution | | *a [*nm] | *σ*_YS_ [MPa] | *σ*_UTS_ [MPa] | *ε*  * *[%] | *E* [Gpa] |
| --- | --- | --- | --- | --- | --- | --- | --- | --- | --- |
|  |  |  | Matrix | Second phase |  |  |  |  |  |
| S1-T | [Ti-Zr_14_]Nb_3_ | Zr_77.78_Ti_5.56_Nb_16.66_ |  | − | 0.3544±0.0002 | 554 | 579 | 16.7 | 49 |
| S1-0.2O | [Ti-Zr_14_]Nb_3_-0.2O | Zr_77.62_Ti_5.55_Nb_16.63_O_0.2_ |  |  | 0.3547±0.0001 | 571 | 581 | 11.6 | 50 |
| S1-0.5O | [Ti-Zr_14_]Nb_3_-0.5O | Zr_77.39_Ti_5.53_Nb_16.58_O_0.5_ |  |  | 0.3556±0.0003 | 629 | 637 | 11.6 | 52 |
| S2-HT1 | [Ti-Zr_8_Hf_6_](Nb_3_) | Zr_44.44_Hf_33.33_Ti_5.56_Nb_16.67_ |  |  | 0.3528±0.0002 | 563 | 700 | 17.4 | 61 |
| S2-0.2O | [Ti-Zr_8_Hf_6_](Nb_3_)-0.2O | Zr_44.35_Hf_33.26_Ti_5.55_Nb_16.64_O_0.2_ |  |  | 0.3530±0.0004 | 672 | 803 | 20.2 | 64 |
| S2-0.5O | [Ti-Zr_8_Hf_6_](Nb_3_)-0.5O | Zr_44.22_Hf_33.16_Ti_5.53_Nb_16.59_O_0.5_ |  |  | 0.3534±0.0001 | 670 | 748 | 6.2 | 64 |
| S3-HT2 | [Ti-Zr_8_Hf_4_Ti_2_](Nb_3_) | Zr_44.44_Hf_22.22_Ti_16.67_Nb_16.67_ |  | − | 0.3455±0.0002 | 704 | 716 | 9.2 | 63 |
| S3-0.5O | [Ti-Zr_8_Hf_4_Ti_2_](Nb_3_)-0.5O | Zr_44.22_Hf_22.10_Ti_16.59_Nb_16.59_O_0.5_ |  | ″ | 0.3460±0.0001 | 796 | 806 | 10.6 | 65 |
| S3-1.0O | [Ti-Zr_8_Hf_4_Ti_2_](Nb_3_)-1O | Zr_44.00_Hf_22.00_Ti_16.50_Nb_16.50_O_1.0_ |  | ″ | 0.3462±0.0003 | 895 | 900 | 10.6 | 69 |
| S3-1.5O | [Ti-Zr_8_Hf_4_Ti_2_](Nb_3_)-1.5O | Zr_43.77_Hf_21.89_Ti_16.42_Nb_16.42_O_1.5_ |  | ″ | 0.3465±0.0002 | 930 | 931 | 18.6 | 67 |
| S3-1.8O | [Ti-Zr_8_Hf_4_Ti_2_](Nb_3_)-1.8O | Zr_43.64_Hf_21.82_Ti_16.37_Nb_16.37_O_1.8_ |  | ″ | 0.3471±0.0001 | 1000 | 1003 | 15.1 | 68 |

**Table S2.** The nominal and measured compositions of all the designed alloys.

| Alloys | Nominal compositions [at. %] | Measured compositions [at. %] |
| --- | --- | --- |
| S1-T | Zr_77.78_Ti_5.56_Nb_16.66_ | Zr_77.90_Ti_5.91_Nb_16.11_O_0.08_ |
| S1-0.2O | Zr_77.62_Ti_5.55_Nb_16.63_O_0.2_ | Zr_78.01_Ti_5.45_Nb_16.30_O_0.24_ |
| S1-0.5O | Zr_77.39_Ti_5.53_Nb_16.58_O_0.5_ | Zr_77.25_Ti_5.68_Nb_16.60_O_0.47_ |
| S2-HT1 | Zr_44.44_Hf_33.33_Ti_5.56_Nb_16.67_ | Zr_44.68_Hf_33.06_Ti_5.78_Nb_16.39_O_0.09_ |
| S2-0.2O | Zr_44.35_Hf_33.26_Ti_5.55_Nb_16.64_O_0.2_ | Zr_44.08_Hf_33.59_Ti_5.44_Nb_16.70_O_0.19_ |
| S2-0.5O | Zr_44.22_Hf_33.16_Ti_5.53_Nb_16.59_O_0.5_ | Zr_44.35_Hf_33.08_Ti_5.59_Nb_16.43_O_0.55_ |
| S3-HT2 | Zr_44.44_Hf_22.22_Ti_16.67_Nb_16.67_ | Zr_44.34_Hf_22.48_Ti_16.53_Nb_16.56_O_0.09_ |
| S3-0.5O | Zr_44.22_Hf_22.10_Ti_16.59_Nb_16.59_O_0.5_ | Zr_44.13_Hf_22.41_Ti_16.32_Nb_16.66_O_0.48_ |
| S3-1.0O | Zr_44.00_Hf_22.00_Ti_16.50_Nb_16.50_O_1.0_ | Zr_43.25_Hf_22.80_Ti_16.28_Nb_16.63_O_1.04_ |
| S3-1.5O | Zr_43.77_Hf_21.89_Ti_16.42_Nb_16.42_O_1.5_ | Zr_43.98_Hf_21.36_Ti_16.95_Nb_16.24_O_1.47_ |
| S3-1.8O | Zr_43.64_Hf_21.82_Ti_16.37_Nb_16.37_O_1.8_ | Zr_43.49_Hf_21.75_Ti_15.89_Nb_16.98_O_1.89_ |

**Table S3.** The damping capacity (tan*δ*)_max_ and corresponding peak temperature *T_p_* of each peak decomposed from the damping curves of the S3-HT2, S3-1.0O, and S3-1.8O alloys.

| Alloys | S3-HT2 | | S3-1.0O | | S3-1.8O | |
| --- | --- | --- | --- | --- | --- | --- |
| Peaks | *T_p_*[K] | (tan*δ*)_max_ | *T_p_*[K] | (tan*δ*)_max_ | *T_p_*[K] | (tan*δ*)_max_ |
| Peak 1 | 474 | 0.0001 | 474 | 0.0005 | 474 | 0.0013 |
| Peak 2 | 575 | 0.0024 | 560 | 0.0041 | 560 | 0.0066 |
| Peak 3 | 675 | 0.0072 | 673 | 0.0077 | 674 | 0.0084 |
| Peak 4 | 745 | 0.0109 | 734 | 0.0119 | 733 | 0.01467 |
| Peak 5 | - | - | 790 | 0.0014 | 790 | 0.0031 |

**Table S4.** Thermal activated temperatures (*T*_r_) for each S-I relaxation process.

| Process | *T*_r_ [K] |
| --- | --- |
| Nb-O | 380 ~ 476 |
| Zr-O | 683 ~ 700 |
| Ti-O | 720 ~ 750 |
| Hf-O | ~753 |

**Table S5.** O solution energy (*E*_O-solu_) and formation energy (*E*_f_) of S3-HT2 alloy with the O atom occupying octahedral interstices of Site 1, Site 2, and Site 3, respectively.

| Site | *E*_O-solu_ [eV] | *E*_f_ [kJ⋅mol^-1^] |
| --- | --- | --- |
| Zr_4_Ti_2_-O (Site 1) | -8.682 | -1.819 |
| Zr_4_Ti_1_Hf_1_-O (Site 2) | -7.517 | -0.279 |
| Zr_4_Ti_1_Nb_1_-O (Site 3) | -7.559 | -0.334 |

**Table S6.** Variation of Bader charge (charge transfer) of oxygen and its neighbored metal atoms in S3-HT2 alloy with the O atom occupying the octahedral interstice of Site 1, Site 2, and Site 3, respectively.

| System | Charge transfer | | | | | | | | | |
| --- | --- | --- | --- | --- | --- | --- | --- | --- | --- | --- |
|  | Ti | Ti | Zr | Zr | Zr | Zr | Hf | Hf | Nb | O |
| Zr_4_Ti_2_ (Site 1) | 0.39 | 0.08 | -0.62 | 0.18 | -0.44 | -0.28 |  |  |  |  |
| Zr_4_Ti_2_-O (Site 1) | 0.11 | 0.22 | 0.28 | 0.64 | 0.73 | 0.56 |  |  |  | -1.37 |
| Zr_4_Ti_1_Hf_1_ (Site 2) | 0.39 |  | 0.1 | 0.07 | -0.77 | 0.12 | 1.16 |  |  |  |
| Zr_4_Ti_1_Hf_1_-O (Site 2) | 0.06 |  | -0.55 | -0.63 | 0.77 | -0.88 | -1.89 |  |  | -1.38 |
| Zr_4_Ti_1_Nb_1_ (Site 3) | -0.4 |  | -0.62 | 0.18 | 0.1 | 0.02 |  |  | 0.89 |  |
| Zr_4_Ti_1_Nb_1_-O (Site 3) | 0.61 |  | 0.73 | 0.84 | -0.51 | 0.64 |  |  | 0.13 | -1.15 |

**Supporting References**

1. C. Dong, Z. J. Wang, S. Zhang, Y. M. Wang, Review of structural models for the compositional interpretation of metallic glasses, *Int. Mater. Rev.* **2020**, *65*, 286-296.
2. C. Pang, B. B. Jiang, Y. Shi, Q. Wang, C. Dong, Cluster-plus-glue-atom model and universal composition formulas [cluster](glue atom)_x_ for BCC solid solution alloys, *J. Alloy. Compd.* **2015**, *652*, 63-69.
3. B. B. Jiang, Q. Wang, C. Dong, P. K. Liaw, Exploration of phase structure evolution induced by alloying elements in Ti alloys via a chemical-short-range-order cluster model, *Sci. Rep.* **2019**, *9*, 3404.
4. A. Tbakeuchi, A. Inoue, Calculations of mixing enthalpy and mismatch entropy for ternary amorphous alloys, *Mater. Trans.* **2000**, *41*, 1372-1378.
5. V. Tuli, P. Burr, Thermodynamic stability of b-phases in Zr-Nb alloys, *Phys. Rev. Mater.* **2023**, *7*, 113607.
6. G. Henkelman, A. Arnaldsson, H. Jónsson, A fast and robust algorithm for Bader decomposition of charge density, *Comput. Mater. Sci.* **2006**, *36*, 354-360.
